# A Distinct DNA Methylation Shift in a Subset of Glioma CpG Island Methylator Phenotypes during Tumor Recurrence

**DOI:** 10.1016/j.celrep.2018.03.107

**Published:** 2018-04-10

**Authors:** Camila Ferreira de Souza, Thais S. Sabedot, Tathiane M. Malta, Lindsay Stetson, Olena Morozova, Artem Sokolov, Peter W. Laird, Maciej Wiznerowicz, Antonio Iavarone, James Snyder, Ana deCarvalho, Zachary Sanborn, Kerrie L. McDonald, William A. Friedman, Daniela Tirapelli, Laila Poisson, Tom Mikkelsen, Carlos G. Carlotti, Steven Kalkanis, Jean Zenklusen, Sofie R. Salama, Jill S. Barnholtz-Sloan, Houtan Noushmehr

**Affiliations:** 1 Department of Neurosurgery, Henry Ford Health System, Detroit, MI 48202, USA; 2 Department of Genetics, Ribeirao Preto Medical School, University of Sao Paulo, Ribeirao Preto, SP, Brazil; 3 Case Comprehensive Cancer Center, Case Western Reserve University School of Medicine, Cleveland, OH 44106, USA; 4 UC Santa Cruz Genomics Institute and Howard Hughes Medical Institute, University of California, Santa Cruz, Santa Cruz, CA 95064, USA; 5 Laboratory of Systems Pharmacology, Harvard Medical School, Boston, MA 02115, USA; 6 Center for Epigenetics, Van Andel Research Institute, Grand Rapids, MI 49503, USA; 7 Laboratory for Gene Therapy, Department of Diagnostics and Cancer Immunology, Greater Poland Cancer Centre, Poznan, Poland; 8 Department of Cancer Immunology, Poznan University of Medical Sciences, Poznan, Poland; 9 International Institute for Molecular Oncology, Poznan, Poland; 10 Department of Pathology and Cell Biology and Neurology Institute for Cancer Genetics, Columbia University, New York, NY 10032, USA; 11 NantOmics, LLC, Santa Cruz, CA, USA; 12 Cure Brain Cancer Biomarkers and Translational Research Laboratory, Prince of Wales Clinical School, UNSW, Sydney, NSW, Australia; 13 Department of Neurosurgery, University of Florida, Gainesville, FL, USA; 14 Department of Surgery and Anatomy, Ribeirao Preto Medical School, University of Sao Paulo, Ribeirao Preto, Brazil; 15 Department of Public Health Sciences, Henry Ford Health System, Detroit, MI 48202, USA; 16 National Cancer Institute, Bethesda, MD 20892, USA; 17 Senior author; 18 Lead Contact

## Abstract

Glioma diagnosis is based on histomorphology and grading; however, such classification does not have predictive clinical outcome after glioblastomas have developed. To date, no *bona fide* biomarkers that significantly translate into a survival benefit to glioblastoma patients have been identified. We previously reported that the *IDH* mutant G-CIMP-high subtype would be a predecessor to the G-CIMP-low subtype. Here, we performed a comprehensive DNA methylation longitudinal analysis of diffuse gliomas from 77 patients (200 tumors) to enlighten the epigenome-based malignant transformation of initially lower-grade gliomas. Intra-subtype heterogeneity among G-CIMP-high primary tumors allowed us to identify predictive biomarkers for assessing the risk of malignant recurrence at early stages of disease. G-CIMP-low recurrence appeared in 9.5% of all gliomas, and these resembled *IDH*-wild-type primary glioblastoma. G-CIMP-low recurrence can be characterized by distinct epigenetic changes at candidate functional tissue enhancers with AP-1/SOX binding elements, mesenchymal stem cell-like epigenomic phenotype, and genomic instability. Molecular abnormalities of longitudinal G-CIMP offer possibilities to defy glioblastoma progression.

## In Brief

*IDH*-mutant lower-grade glioma glioblastoma often progresses to a more aggressive phenotype upon recurrence. de Souza et al. examines the intra-subtype heterogeneity of initial G-CIMP-high and use this information to identify predictive biomarkers for assessing the risk of recurrence and malignant transformation.

## Graphical Abstract

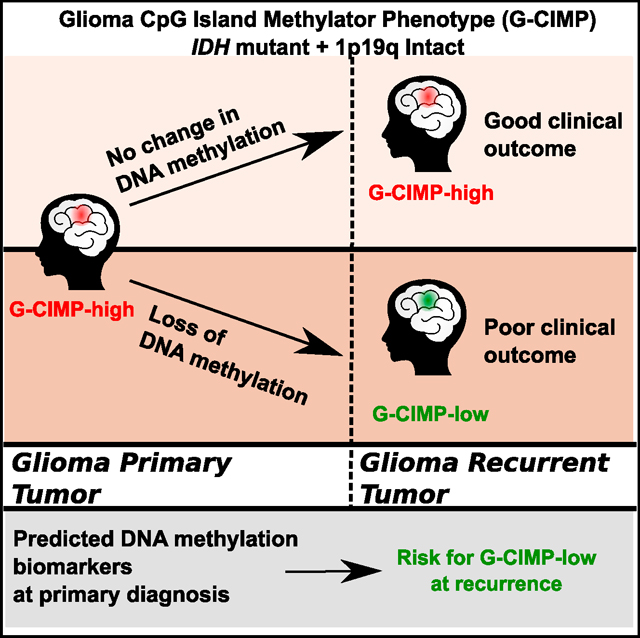

## INTRODUCTION

Heterozygous gain-of-function mutations in *IDH1/2* (isocitrate dehydrogenase (NADP(+) 1/2; IDH) is traditionally a hallmark of a subset of gliomas associated with favorable patient outcomes (Parsons et al., 2008; Yan et al., 2009). Mutant IDH protein produces the oncometabolite D-2-hydroxyglutarate (2HG), which may establish the glioma-CpG island methylator phenotype (G-CIMP) (Noushmehr et al., 2010) by presumably extensive remodeling of the tumor methylome (Turcan et al., 2012). The incorporation of *IDH* mutation status into the classical histopathology and grading system by the updated 2016 World Health Organization (WHO) classification of tumors of the CNS represents an emerging concept in which diagnosis of diffuse gliomas should be structured and refined in the molecular taxonomy era (Louis et al., 2016; Malta et al., 2017). Although *IDH* mutation is retained upon glioma recurrence (Bai et al., 2016; Mazor et al., 2015), mutant *IDH1* may convert from driver to passenger (Johannessen et al., 2016), and, in some patients, neither mutant *IDH1* nor the oncometabolite 2HG are strictly required for clonal expansion at recurrence (Mazor et al., 2017).

Glioblastoma (GBM) is a highly aggressive brain cancer and accounts for 46.6% of primary malignant brain tumors with a 5-year overall survival estimate post-diagnosis of 5.5% (Ostrom et al., 2016). The WHO histomorphology and grading classification of diffuse gliomas does not have predictive clinical outcomes after GBMs have developed (Louis et al., 2016; Sanai et al., 2011). Treating initially lower-grade glioma (LGG) that relapses and undergoes malignant transformation to GBM is one of the greatest challenges in neuro-oncology (Stupp et al., 2005, 2009). To date, despite the efforts of the neuro-oncology community, no treatment regimens or *bona fide* biomarkers that significantly translate into a survival benefit to GBM patients have been identified.

Widespread genetic alterations of high-grade gliomas have been extensively examined. Mutational branching models’ assumption of divergence time in GBM suggested that recurrence-associated clones diverged from untreated clones years before diagnosis (Wang et al., 2016). The mutational landscape of multisector and/or long-term recurrent malignant glioma biopsies can inform therapy-driven evolution and personalized targeted therapies in GBM (Johnson et al., 2014; Kim et al., 2015; Lee et al., 2017; Wang et al., 2016). Frequent genomic chromothripsis events and the later acquired DNA mismatch repair deficiency by GBM cells may positively select for treatment-resistant clones (Erson-Omay et al., 2017).

Epigenetics refers to differential control of gene expression and alternate cellular phenotypes that are not coded in the individual’s DNA sequence but, rather, determined by chromatin structure, particularly via covalent modifications of DNA (DNA methylation) and histone proteins (Sharma et al., 2010). Epigenetically based molecular classification of 932 adult diffuse primary gliomas (WHO grades II to IV) analyzed by our group uncovered the existence of three cohesive molecular subtypes of IDH mutant gliomas (Codel, G-CIMP-high, and G-CIMP-low) and four subtypes of *IDH*-wild-type gliomas (classic-like, mesenchymal-like, LGm6-GBM, and pilocytic astrocytoma [PA]-like) with characteristic patient outcomes. Accordingly, IDH mutant non-Codel DNA methylation signatures allowed the segregation of LGG-GBM G-CIMP tumors into two discrete disease subtypes independent of neuropathological grading (G-CIMP-high and G-CIMP-low). The G-CIMP-low subtype accounts for 6% of all IDH mutant diffuse primary gliomas and is characterized by lower levels of DNA methylation at specific CpG signatures and an unfavorable overall survival relative to the G-CIMP-high subtype, which accounts for 55% of all IDH mutant diffuse primary gliomas (Ceccarelli et al., 2016). By evaluating a small cohort of matched primary and recurrent diffuse gliomas, we recently reported that the G-CIMP-high subtype would be a predecessor to the G-CIMP-low subtype, which suggested a disease progression model relative to G-CIMP (Ceccarelli et al., 2016). However, the critical question of whether the spatial and temporal dynamics of epimethyl patterns of G-CIMP offer new possibilities for assessing the risk of malignant recurrence at early stages of glioma evolution to defy glioma progression remains unresolved. Comprehensive evolution of initially LGG G-CIMP methylomes throughout the course of cell-malignant transformation to GBMs has potential clinical implications for identifying predictive biomarkers to abrogate the establishment, recurrence, and progression of a malignant glioma phenotype.

## RESULTS

### Samples and Clinical Data

A summary of clinical data is represented in Tables 1 and 2 and reflects our effort to manually update the available information at The Cancer Genome Atlas (TCGA) Biospecimen Core Resource (BCR) combined with published datasets (Mazor et al., 2015; Bai et al., 2016; Mazor et al., 2017) and our own cohort with known *IDH* mutation and 1p-19q (short arm of chromosome 1 and long arm of chromosome 19) co-deletion status. The majority of samples were *IDH* mutant non-Codel at primary (54 of 74; 72.97%) and first recurrence (50 of 69; 72.46%) surgery time points. Stratification of histology and grading among the *IDH* mutant non-Codel cases included astrocytoma grade II as primary (47; 87.04%) as well as anaplastic astrocytoma grade III (18; 36%) and glioblastoma grade IV (19; 38%) at first recurrence.

### Spatial and Temporal Epimethyl Pattern Dynamics of Evolution in Adult Diffuse Longitudinal Gliomas

Our group and others reported the widespread differences in DNA methylation in adult diffuse primary gliomas (Sturm et al., 2012; Brat et al., 2015; Ceccarelli et al., 2016). We previously grouped primary gliomas into two *IDH*-driven macro-clusters eventually leading to the identification of three *IDH* mutant-specific DNA methylation subtypes (Codel, G-CIMP-high, and G-CIMP-low) and three *IDH* wild-type-specific DNA methylation subtypes (classic-like, mesenchymal-like, and LGm6). Based on the molecular similarity with PAs, LGG tumors classified as LGm6 pan-glioma DNA methylation subtype were further labeled as PA-like. Additionally, the GBMs falling into this group were best described as LGm6-GBM for their original pan-glioma DNA methylation cluster and tumor grade (Ceccarelli et al., 2016).

**Figure 1.**
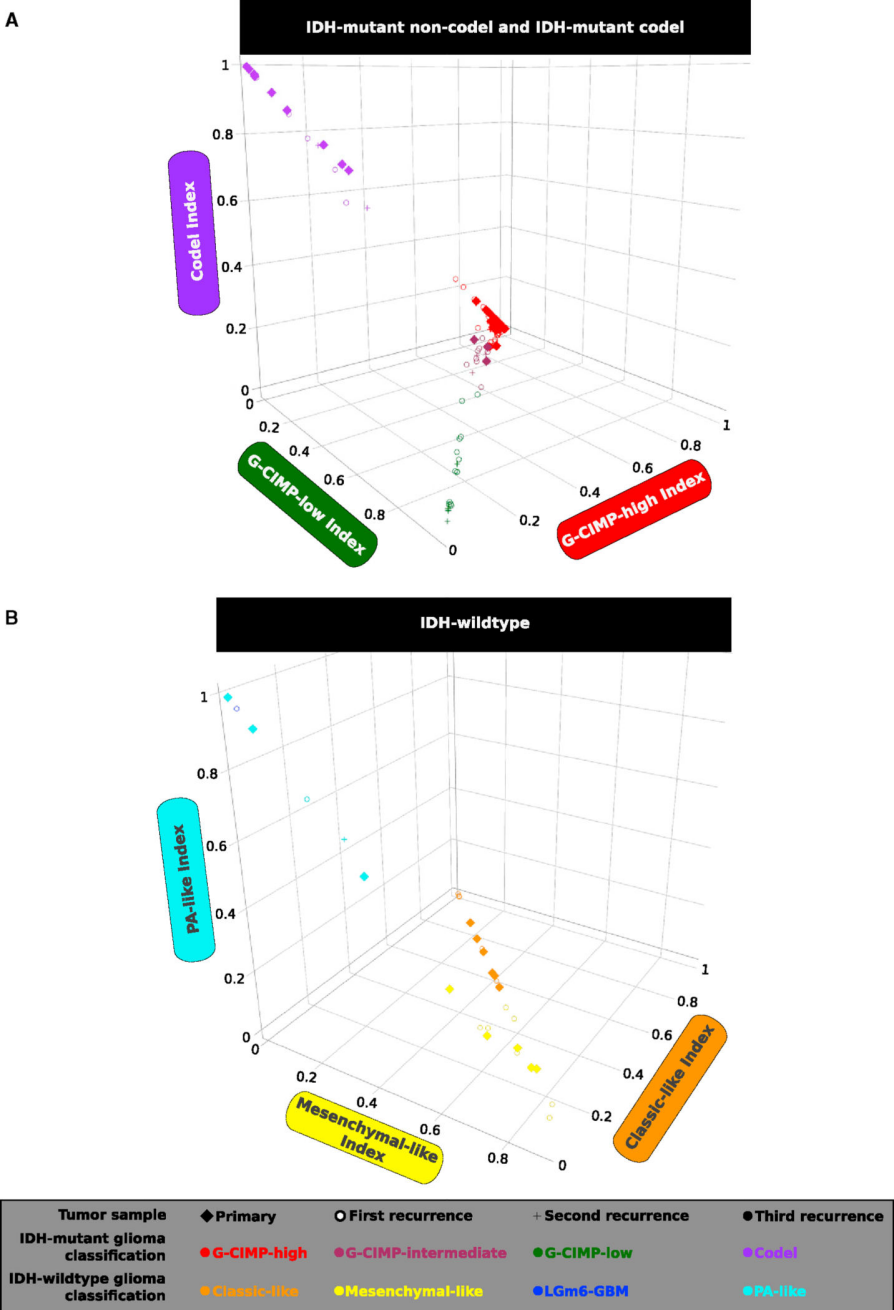
Identification of Longitudinal Tumors with a G-CIMP-High to G-CIMP-Low Epigenetics Shift during Recurrence and Malignant Tumor Progression The methylomes of 200 longitudinally collected TCGA and non-TCGA adult diffuse gliomas (grades II to IV) from 77 patients profiled on the 450,000 platform were classified by supervised random forest (RF) computational approaches into one of the 7 pan-glioma DNA methylation subtypes (accuracy > 95% on average) using the CpG probe signatures described in Ceccarelli et al. (2016). (A) This 3D scatterplot using IDH mutant Codel (negative control of G-CIMP signatures) and IDH mutant non-Codel G-CIMP-high and G-CIMP-low indices predicted by the RF model shows a distinct set of samples within the IDH mutant non-Codel G-CIMP subtypes exhibiting relatively intermediate DNA methylation profiles. This subgroup of samples has been named G-CIMP-intermediate post-RF assessment. A subset of initially LGG G-CIMP-high tumors switches to a G-CIMP-low phenotype at first recurrence, whereas a subset of tumors retains their original G-CIMP-high phenotype at first recurrence as a form of epigenetic memory. (B) 3D scatterplot using *IDH*-wild-type PA-like, classic-like, and mesenchymal-like indices predicted by RF shows that *IDH*-wild-type gliomas do not change significantly in terms of their DNA methylation patterns during disease relapse.

TCGA adult diffuse glioma samples not classified in our published analysis (n = 39, 9 primary and 30 recurrent), in addition to 20 primary cases previously included, were classified into one of the 7 DNA methylation subtypes. To do this, we applied a random forest (RF) machine learning prediction model using our defined DNA methylation probe signatures described in Ceccarelli et al. (2016): *IDH* mutant tumor-specific (n = 1,308), *IDH* mutant subtypes (n = 163), and *IDH*-wild-type tumor-specific (n = 914). We extended our analysis by similarly assigning each tumor sample in the non-TCGA published longitudinal cohorts (Mazor et al., 2015, 2017, n = 81; Bai et al., 2016, n = 48) to one of the DNA methylation subtypes. Additionally, we profiled and classified a total of 12 primary and recurrent glioma samples generated from our own cohort and predicted the *IDH* and 1p-19q statuses of 9 tumor fragments derived from biopsies of 3 distinct patients (Tables S1 and S2). To do this, we integrated an additional set of 1,300 tumor-specific probes that discriminated the pan-glioma primary cohort into two macro groups: the LGm1/LGm2/LGm3 DNA methylation macro group harboring the *IDH1* or *IDH2* mutation versus the LGm4/LGm5/LGm6 DNA methylation macro group comprising glioma samples carrying *IDH*-wild-type (Ceccarelli et al., 2016). Therefore, we examined the spatial and temporal dynamics of DNA methylomes of 200 longitudinally collected TCGA and non-TCGA gliomas from 77 patients profiled on the Illumina HumanMethylation450 bead arrays (450,000) platform. Of the 200 glioma fragments, 132 (66%) were classified as G-CIMP-high, 20 (10%) were classified as Codel, 19 (9.5%) were classified as G-CIMP-low, 12 (6%) were classified as mesenchymal-like, 11 (5.5%) were classified as classic-like, 5 (2.5%) were classified as PA-like, and 1 (0.5%) was classified as LGm6-GBM by supervised RF computational approaches with high specificity and sensitivity (accuracy > 95% on average) (Figures 1A and 1B; Table S1).

**Figure 2.**
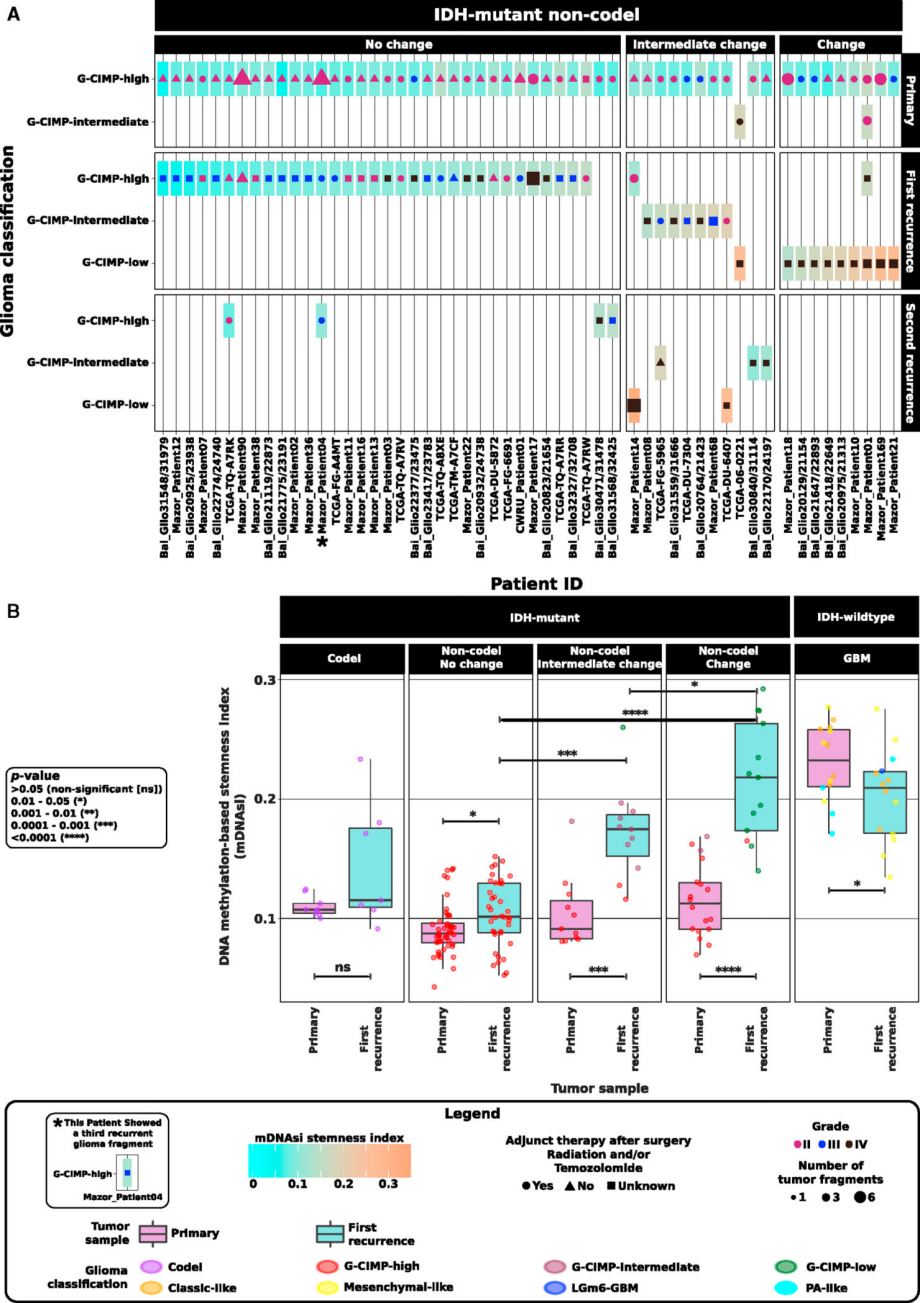
Acquisition of an *IDH*-Wild-Type and Stem Cell-like GBM Phenotype by G-CIMP-Low at Recurrence An overview of the longitudinal glioma cohort (n = 77 patients) across all tissue source sites is shown and highlights the stratification of glioma patients according to the temporal epigenomic profile dynamics of their tumors from initial (primary) diagnosis to first recurrent disease. (A) A subset within the IDH mutant non-Codel G-CIMP-high subtype that retains their original epigenomics phenotype at first recurrent disease (o change), a subset within the IDH mutant non-Codel macro group manifesting the G-CIMP-intermediate DNA methylation profile at primary and/or recurrent diseases plus a subset within the IDH mutant non-Codel macro group exhibiting the G-CIMP-low phenotype at second recurrence (these patients are collectively defined as those showing intermediate changes in their epigenomic profiles), and a subset within the IDH mutant non-Codel macro group (n = 9 patients) showing a dramatic epigenomic shift toward malignant transformation from G-CIMP-high at primary to G-CIMP-low at first recurrence (change). Adult diffuse longitudinal gliomas are categorized according to their stem cell-like prevalence/degree of undifferentiation (stemness) by using the DNA methylation-based stemness index (mDNAsi) as relative metric (a score value from 0 to 1). Each box represents a patient tumor colored according to its mDNAsi at primary and recurrent stages of the disease. When multiple tumor fragments are available per surgical resection, mDNAsi represents an average value of geographically distinct tumor pieces derived from the same patient surgery. Symbol color, size, and shape within each box represent tumor grade, the number of tumor fragments, and adjunct therapy (radiation and/or TMZ) received after surgery of primary and recurrent tumors. (B) G-CIMP-low first recurrent tumors possess a higher overall stemness index in relation to their G-CIMP-high primary counterparts and G-CIMP-high first recurrent tumors. The landscape of the stemness index in G-CIMP-low first recurrent tumors highly resembles those found in *IDH*-wild-type primary GBMs. See also Figure S1.

Despite harboring the *IDH* mutation, primary tumors that belong to the G-CIMP-low subtype were reported to have lower DNA methylation levels and worse clinical outcomes in relation to primary tumors that belong to the G-CIMP-high subtype (Ceccarelli et al., 2016). A 3D scatterplot using G-CIMP-low and G-CIMP-high indices predicted by the RF model (Figure 1A) allowed us to visualize the phenotypic relationships of G-CIMP-positive longitudinal tumors, suggesting a distinct set of samples within the IDH mutant non-Codel G-CIMP subtypes that showed relatively intermediate DNA methylation profiles at a G-CIMP-low index threshold of < 0.5 and R 0.2 and at a G-CIMP-high index threshold of R 0.5 and < 0.75. We named this subgroup of samples G-CIMP-intermediate post-RF assessment (i.e., n = 3 primary, n = 8 first recurrent, and n = 3 second recurrent tumor fragments derived from 11 distinct patients). The G-CIMP-intermediate subgroup was characterized by a modest degree of DNA methylation changes trending toward the G-CIMP-low subtype (Figures 1A and 2A; Table S1). This may suggest that G-CIMP-intermediate reflects an early-stage transition from G-CIMP-high to G-CIMP-low. Notably, we demonstrated a dramatic epigenomic shift toward malignant progression from G-CIMP-high at primary to G-CIMP-low at first recurrence in 9 patients (Figure 2A). Although all G-CIMP-low tumors at first recurrence were grade IV, not all grade IV tumors transitioned to G-CIMP-low, suggesting that grade may not be the only indicator of G-CIMP-low progression (Figure 2A). We did not observe any significant changes in the *IDH* mutant Codel and *IDH*-wild-type glioma subtypes in terms of their epigenomic profile toward recurrent disease (Figures S1A and S1B; Table S1).

### Acquisition of an *IDH*-Wild-Type and Stem Cell-like GBM Phenotype by G-CIMP-Low at Recurrence

G-CIMP-low primary tumors showed a molecular signature associated with a stem cell-like phenotype at DNA binding motifs for SOX transcription factors (TFs) with the worst overall clinical outcomes within the IDH mutant non-Codel genotype (Ceccarelli et al., 2016). To explore the relationship of stemness and G-CIMP malignant transformation, we applied the DNA methylation-based stemness index (mDNAsi) to categorize our adult diffuse glioma longitudinal cohort according to its degree of undifferentiation as a function of glioma malignancy (Figures 2A and 2B). Briefly, mDNAsi is a score value resulting from a concat-enation of three stemness signatures (a total of 219 probes) mainly defined by using 450,000 DNA methylation profiles and a one-class logistic regression predictive model (Malta et al., 2018) on human stem/progenitor cells and their differentiated progeny from the Progenitor Cell Biology Consortium (PCBC) (Daily et al., 2017; Salomonis et al., 2016). mDNAsi ranges from zero to one and provided a relative metric to classify a total of 9,627 TCGA samples across 33 distinct tumor types according to their stem cell-like prevalence (stemness). mDNAsi was able to recapitulate known features of stemness in the *IDH*-wild-type, mesenchymal-like, and classic-like subtypes (Malta et al., 2018). Therefore, mDNAsi applied to our longitudinal G-CIMP progression model provided an independent relative metric to estimate progression in gliomas independent of grade and known overall survival predictors (Figures 2A and 2B). We showed that *IDH*-wild-type primary and first recurrent tumors had the highest overall stemness index (medians of 0.23 and 0.2, respectively) compared with the entire *IDH* mutant cohort at primary and first recurrence (medians of 0.1 and 0.14, respectively); however, the degree of stemness within *IDH*-wild-type shifted from primary to first recurrence (p = 0.05) (Figure 2B), suggesting that *IDH*-wild-type recurrent gliomas may be defined by expansion of a resistant clone that is more differentiated yet aggressive in nature, as reported for metastatic melanoma cells (Cheli et al., 2011). Interestingly, G-CIMP-low first recurrent tumors showed a higher overall stemness index in relation to their G-CIMP-high primary counterparts (p < 0.0001; medians of 0.22 and 0.1, respectively). Compared with G-CIMP-high first recurrent tumors, the landscape of the stemness index in G-CIMP-low first recurrent tumors highly resembled those found in *IDH*-wild-type primary GBMs (p < 0.0001; medians of 0.1 and 0.2, respectively) (Figure 2B). Therefore, we defined a subset of G-CIMP-low tumors that acquire a stem cell-like phenotype upon first recurrence, suggesting that a stem cell-like aggressive tumor behavior may exist within IDH mutant non-Codel and contribute to their resistance to adjuvant therapy and relapse as the G-CIMP-low phenotype (Figure 2A).

### Evolution of G-CIMP-Low Methylomes Resembles a Signature toward Mesenchymal Transformation

*IDH*-wild-type GBMs are highly aggressive brain tumors because of a small subpopulation of cancer stem cells capable of tumor initiation *in vivo* and multi-lineage differentiation potential to support therapeutic resistance, recurrence, and the progressive growth of tumors (Lathia et al., 2015; Singh et al., 2004; Vescovi et al., 2006). G-CIMP tumors were found to belong to the proneural gene expression subtype of gliomas (Noushmehr et al., 2010; Verhaak et al., 2010), and, interestingly, a mathematical model of GBM evolution suggested that most non-G-CIMP mesenchymal GBMs evolve from a proneural-like precursor downstream of chromosome (chr) 7 gain and chr10 loss, followed by *CDKN2A* loss and/or *TP53* mutation (Ozawa et al., 2014). A subtype transition from proneural to the aggressive GBM mesenchymal pattern was documented upon therapy resistance and re-occurrence of the disease (Bhat et al., 2013; Phillips et al., 2006). In line with this, we sought to determine whether the acquisition of an *IDH*-wild-type stem cell-like GBM phenotype by G-CIMP-low at first recurrence showed molecular similarity to mesenchymal cell differentiation.

**Figure 3.**
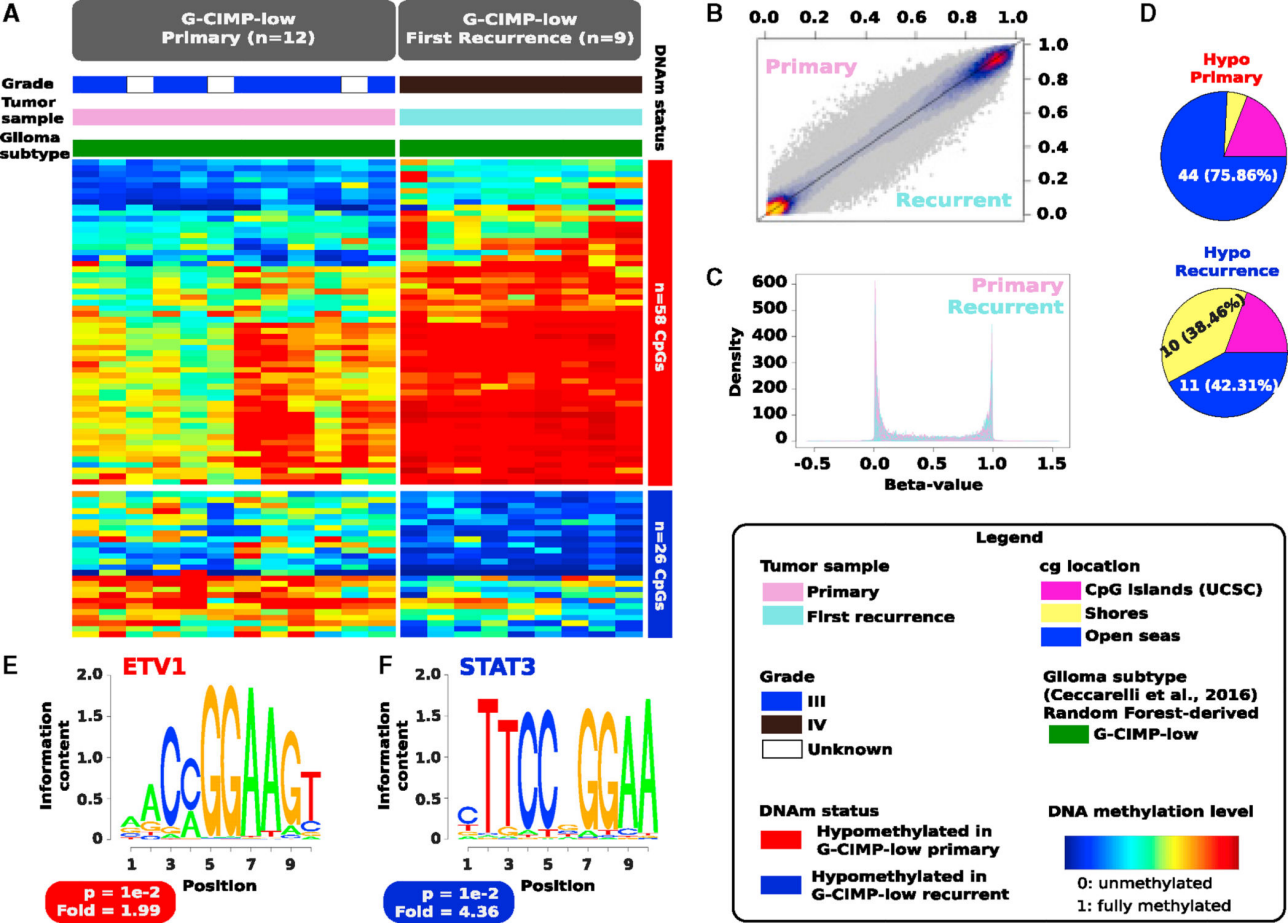
Evolution of G-CIMP-Low Methylomes Resembles a Signature toward Mesenchymal Transformation (A) Heatmap of DNA methylation data. Columns represent *de novo* (primary) G-CIMP-low tumors (n = 12) and acquired (first recurrent) G-CIMP-low tumors (n = 9) sorted by hierarchical clustering. Rows represent CpG probes identified as differentially methylated after supervised analysis between de novo and acquired G-CIMP-low tumors. Fifty-eight hypomethylated CpGs define the G-CIMP-low primary methylomes, whereas 26 hypomethylated CpGs define the G-CIMP-low recurrent methylomes (FDR < 0.05, absolute difference in mean methylation beta value > 0.2). The labels at the top of the heatmap represent clinical and molecular features of interest. The saturation of either color scale reflects the magnitude of the difference in DNA methylation level. (B and C) This 2D scatterplot (B) and density plots (C) of 450,000 probes show that G-CIMP-low methylomes share epigenome-wide features at primary and first recurrent diseases. (D) Genomic distribution of hypomethylated CpGs (n = 84) that distinguish the G-CIMP-low primary and first recurrent methylomes. (E and F) *De novo* (primary) (E) and acquired (first recurrent) (F) G-CIMP-low methylomes are defined by DNA signature motifs for ETV1 and STAT3, respectively, known to play a role as master regulators of mesenchymal lineage differentiation.

Supervised analysis of DNA methylation of *de novo* (primary) G-CIMP-low (n = 12) and acquired (first recurrent) G-CIMP-low (n = 9) showed that, even though these tumors shared epigenome-wide features, G-CIMP-low primary and recurrent methylomes were distinguished by 84 differentially methylated probes (58 hypomethylated CpGs in G-CIMP-low primary and 26 hypomethylated CpGs in G-CIMP-low recurrent, false discovery rate [FDR] < 0.05, absolute difference in mean methylation beta value > 0.2) (Figures 3A–3C; Table S3). Most of the hypomethylated probes at primary (44, 75.86%) were located within intergenic regions known as open seas, whereas the hypomethylated probes at first recurrence were equally distributed between genomic regions of 2,000 bp upstream and downstream, flanking CpG island (CGI) boundaries known as shores (10, 38.46%) and intergenic open sea regions (11, 42.31%) (Figure 3D).

In-depth known motif analysis of G-CIMP-low methylomes led to the identification of DNA signature motifs for ETV1 (5ʹ-AACCG GAAGT-3ʹ) at CpG sites hypomethylated in G-CIMP-low primary and STAT3 (5ʹ-CTTCCGGGAA-3ʹ) at CpG sites hypomethylated in G-CIMP-low recurrent (geometric test; p = 1e—2; fold enrichment of 1.99 and 4.36, respectively) (Figures 3E and 3F). STAT3 is known to be the master regulator of mesenchymal differentiation in glioma cells (Carro et al., 2010), and, hence, this provides meaningful insights into the evolution of G-CIMP-low recurrent cells along the aberrant mesenchymal lineage transformation and into the unfavorable patient outcomes because these tumors can emerge as secondary GBMs. ETV1 oncoprotein was reported to induce the epithelial-to-mesenchymal transition (EMT)-like metastatic progression and increased invasiveness/aggressiveness of gastric adenocarcinomas by upregulation of *SNAIL* expression, a classical EMT driver gene (Li et al., 2013).

Therefore, the establishment of a G-CIMP-low methylome in primary gliomas may occur in a cell-intrinsic manner, whereas the establishment of a G-CIMP-low methylome in malignant recurrent gliomas may reflect the response of transformed cells to epigenetics-selective pressure in the tumor microenvironment, possibly a response to therapy. This provides the lay basis to not only support the notion that G-CIMP-low at primary and G-CIMP-low at recurrence can be considered two separate tumor entities but also to hypothesize that, despite heterogeneous molecular alterations, epigenetic events during G-CIMP-low evolution converged toward the aberrant mesenchymal lineage transformation at primary and recurrence.

### G-CIMP-High to G-CIMP-Low Malignant Transformation Is Defined by Epigenomic Changes at Genomic Biofeatures Associated with Glioma Progression and Normal Development

**Figure 4.**
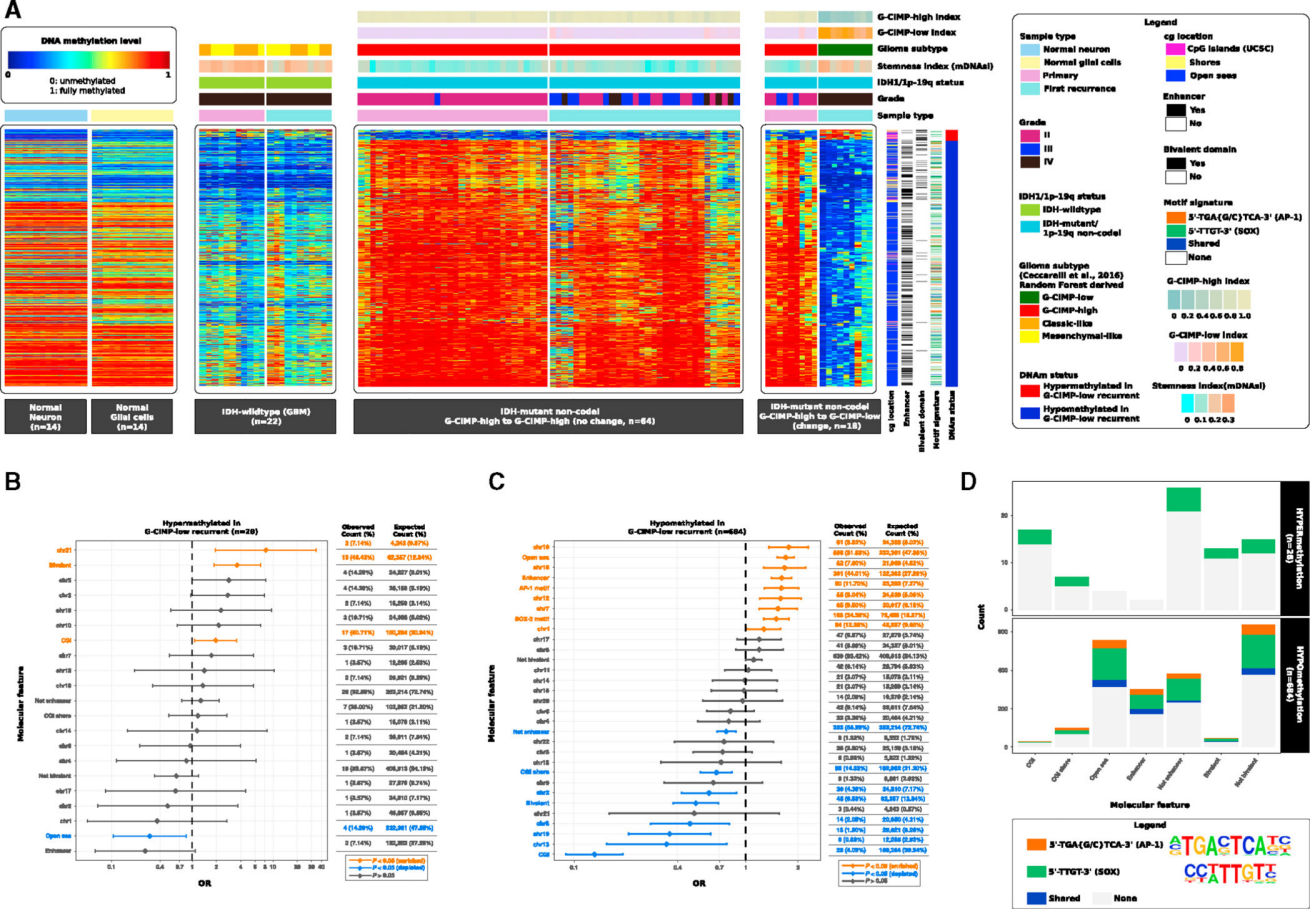
G-CIMP-High to G-CIMP-Low Malignant Transformation Is Defined by Epigenomic Changes at Genomic Biofeatures Associated with Glioma Progression and Normal Development (A) Heatmaps of DNA methylation data. Columns represent non-tumor brain cells (normal neuron cells and normal glial cells, n = 28), *IDH*-wild-type GBMs (n = 22), and IDH mutant non-Codel gliomas (n = 82) grouped according to their epigenomic profiles at primary and first recurrent surgery time points. Normal and tumor samples are sorted by hierarchical clustering. Rows represent CpG probes identified after supervised analysis between DNA methylation of G-CIMP-high tumors at primary diagnosis and their G-CIMP-low counterparts at first recurrence sorted by hierarchical clustering (n = 28 hypermethylated probes and n = 684 hypomethylated probes in G-CIMP-low first recurrent tumors; FDR < 0.05, difference in mean methylation beta value < —0.4 and > 0.5). Labels at the top and tracks on the right of the heatmaps represent clinical and molecular features of interest. The saturation of either color (scale from blue to red) reflects the magnitude of the difference in DNA methylation level. (B and C) OR for the frequencies of differentially hypermethylated probes (B) and differentially hypomethylated probes (C), respectively, that overlap a particular molecular feature relative to the expected genome-wide distribution of 450,000 probes. (D) *De novo* and known motif scan analyses identified recurring patterns in DNA that are presumed to have sequence binding-specific sites for the c-JUN/AP-1 (5ʹ-TGA{G/C}TCA-3ʹ) and SOX family of transcription factors (5ʹ-TTGT-3ʹ). The molecular features overlapping both motif signatures are shown. See also Figures S2 and S3.

We performed a supervised analysis to determine distinct epigenetic changes between the groups defined by a glioma subtype shift (Figures 2A and S1; Table S1). We did not identify any significant epigenetic difference between *IDH*-wild-type primary and recurrent gliomas and *IDH* mutant Codel primary and recurrent gliomas (Figures S1A and S1B; Table S1). Using a core set of 9 cases that significantly shift their DNA methylation patterns from G-CIMP-high at initial (primary) diagnosis to G-CIMP-low at first recurrence (Figure 2A; Table S1), we identified 684 differentially hypomethylated CpG probes and 28 differentially hypermethylated CpG probes (FDR < 0.05, difference in mean methylation beta value > 0.5 and < –0.4) associated with G-CIMP-low recurrence (Figure 4A; Table S4). When we compared these 712 G-CIMP-low signatures at recurrence with non-tumor, normal neuronal cells and normal glial cells, we observed that the G-CIMP-high (primary and first recurrent) tumors were normal-like, contrary to what we found for G-CIMP-low recurrent tumors and grade IV *IDH*-wild-type (primary and recurrent) GBMs. Therefore, the 712 G-CIMP-low recurrent CpG signatures were able to stratify IDH mutant non-Codel G-CIMP tumors exhibiting progressed disease and highly aggressive (*IDH* wild-type-like) phenotypes (Figure 4A). This finding (Figure 4A), combined with our stemness and G-CIMP- low evolution analyses (Figures 2 and 3), demonstrated that G-CIMP-low recurrent tumors shared epigenetic characteristics with *IDH*-wild-type primary GBMs. Although all G-CIMP-low tumors were classified as grade IV (10 of 20 *IDH* mutant GBMs at first recurrence, 50%), not all IDH mutant grade IV first recurrent gliomas progressed to the G-CIMP-low phenotype; in fact, 35% (7 of 20) of grade IV *IDH* mutant gliomas at first recurrence were classified as G-CIMP-high, whereas 15% (3 of 20) were classified as G-CIMP-intermediate tumors (Figure 2A). To evaluate whether there were differences within grade IV G-CIMPs, we performed a supervised DNA methylation analysis between grade IV G-CIMP-low at first recurrence (n = 9, change) and grade IV G-CIMP-high at first recurrence (n = 6, no change). We observed 350 differentially methylated probes (Wilcoxon rank-sum test, p < 0.01; difference in mean methylation beta value < —0.4 and > 0.5; Figure S2). Collectively, these findings suggest that G-CIMP-high to G-CIMP-low follows an alternative epigenetic roadmap toward disease relapse independent of grade (Figures 2, 3, 4A, and S2).

CpG sites exhibiting DNA hypermethylation in G-CIMP-low at first recurrence were significantly enriched for CGIs (odds ratio [OR] = 1.96, 95% confidence interval [CI]: 1.07–3.58), bivalent chromatin domains (OR = 3.61, 95% CI: 1.87–6.98), and chr21 (OR = 8.17, 95% CI: 1.95–34.32) (Figure 4B, p < 0.05 [enriched]). We also observed a depletion of probes positioned within intergenic regions or open seas (OR = 0.30, 95% CI: 0.10–0.85) (Figure 4B, p < 0.05 [depleted]). Markedly, CpG sites showing DNA hypomethylation in G-CIMP-low at first recurrence were significantly enriched for open seas (OR = 1.70, 95% CI: 1.52–1.91), enhancer elements (OR = 1.61, 95% CI: 1.41–1.85), and chr1, chr7, chr10, chr12, and chr16 (OR > 1.0) (Figure 4C, p < 0.05 [enriched]). However, we observed a depletion of probes located at CGIs (OR = 0.13, 95% CI: 0.09–0.19), shores (OR = 0.67, 95% CI: 0.55–0.83), bivalent chromatin domains (OR = 0.51, 95% CI: 0.38–0.69), non-enhancer elements (OR = 0.77, 95% CI: 0.68–0.87), and chr2, chr8, chr13, and chr19 (OR < 0.3) (Figure 4C, p < 0.05 [depleted]).

Genomic abnormalities pertaining to chromosomes 1, 7, 10, 12, and 19 were documented in gliomas (Brennan et al., 2013; Brat et al., 2015). Chromothripsis events affecting chr1, chr7, and chr12 with a high level of amplification were found in high-grade gliomas (Erson-Omay et al., 2017). Recently, a functional study showed that chr7 gain is a repeated genomic event in glioma stem cell lines from primary and multiple sections of tumors at recurrence in a GBM patient, which correlated to the tissue-wide expansion of a new clone in the recurrent tumor (Baysan et al., 2017). Taken together, these findings suggest that chromosomal alterations may contribute down the road of tumor evolution in a rare subgroup of LGG CIMP gliomas progressing to GBMs.

The majority of CpG sites that underwent a massive DNA demethylation in G-CIMP-low relapsed tumors were primarily found within intergenic open sea regions (558 of 684, 81.58%) (Figures 4A and 4C), a finding consistent with our previous study of primary G-CIMP-high and primary G-CIMP-low tumors (Ceccarelli et al., 2016). By aggregating chromatin hidden Markov model (chromHMM) data from the NIH Roadmap Epigenomics Consortium (Kundaje et al., 2015) with the 712 differentially methylated regions (DMRs) identified in G-CIMP-low progressed tumors, we observed these genomic elements to be functionally relevant in defining differentiated adult tissue phenotypes and pluripotency in stem cells (Figure S3). Loss of CpG methylation at these known functional genomic elements associated with normal development and pluripotency defines a possible mechanism of glioma progression that may lead to improved targeted therapy against the G-CIMP-low tumor phenotype.

DNA methylation signatures of multiple disease-related genes and intergenic regions have been related to mortality outcomes (Zhang et al., 2017), providing evidence for the collaborative role of DNA methylation and non-coding functional regions in the modulation of cell phenotypes. For a more functional view of the recurring patterns in hypomethylated DNA that are presumed to have sequence binding-specific sites for TFs implicated in tumor relapse and progression to G-CIMP-low (n = 684 CpG sites), we performed de novo and known DNA motif scan analyses. The top ranked *de novo* motif signature, 5ʹ-TGA{G/C} TCA-3ʹ (geometric test, p = 1e—16, fold enrichment = 3.04), corresponded to known motifs associated with the TFs JUN/AP-1 (geometric test, *q* = 0, fold enrichment = 2.86), FOSL2 (geometric test, *q* = 0, fold enrichment = 2.42), FRA1 (geometric test, *q* = 0, fold enrichment = 2.01), BATF (geometric test, *q* = 2e-4, fold enrichment = 1.72), ATF3 (geometric test, *q* = 4e-4, fold enrichment = 1.67), and AP1 (geometric test, *q* = 7e-4, fold enrichment = 1.60). AP-1 (activating protein-1) is a collective term referring to homodimeric or heterodimeric TFs composed of basic region-leucine zipper (bZIP) protein JUN, FOS, or ATF subfamilies. AP-1 is involved in cellular proliferation, transformation, and death (Shaulian and Karin, 2002). We found that AP-1 may significantly bind to probes of demethylated DNA (80 of 684, 11.70%) in G-CIMP-low-progressed cases (OR = 1.61, 95% CI: 1.28–2.03) (Figure 4C). From the list of 684 hypomethylated regions, we then extracted those that mapped to the DNA motif signature 5ʹ-TGA{G/C}TCA-3ʹ (n = 87 DMRs). Among them, 87.36% were located in open seas, and 59.77% overlapped with enhancers known to define tissue phenotypes (76 and 52 DMRs, respectively) (Figures 4A and 4D).

Our findings also suggested the motif signature 5ʹ-TTGT-3ʹ, known to be associated with SOX family members of TFs, to be significantly enriched: SOX3 (geometric test, *q* = 0, fold enrichment = 1.49), SOX6 (geometric test, *q* = 0, fold enrichment = 1.47), SOX2 (geometric test, *q* = 1e—4, fold enrichment = 1.67), SOX10 (geometric test, *q* = 7e—4, fold enrichment = 1.39), SOX4 (geometric test, *q* = 2.2e—3, fold enrichment = 1.55), and SOX15 (geometric test, *q* = 2.2e—3, fold enrichment = 1.47). We observed that SOX TFs collectively may bind to 226 differentially hypomethylated regions, most of them located in intergenic open sea regions (200 of 226, 88.50%) (Figures 4A–4D), thus recapitulating epigenetic features of G-CIMP-low primary tumors (Figures 3B–3D; Ceccarelli et al., 2016). Additionally, a set of 5 differentially DNA hypermethylated regions, mostly located in CGIs, showed DNA binding sites for the SOX-related motif signature (Figures 4A–4D). Thirty-five of 684 hypomethylated DNA regions (5.12%) shared both the 5ʹ-TGA{G/C}TCA-3ʹ and 5ʹ-TTGT-3ʹ motif signatures (Figure 4D). Therefore, the above results suggest that DNA demethylation events at CpGs deregulated in G-CIMP-low at first recurrence would alter functional enhancers and DNA binding sites recognized by c-JUN/AP-1, contributing to G-CIMP progression toward a GBM-like phenotype (compilation of results shown in Figure 4).

### Predictive Biomarker Signatures Can Predict the Risk for G-CIMP-Low Progression at Primary Diagnosis

**Figure 5.**
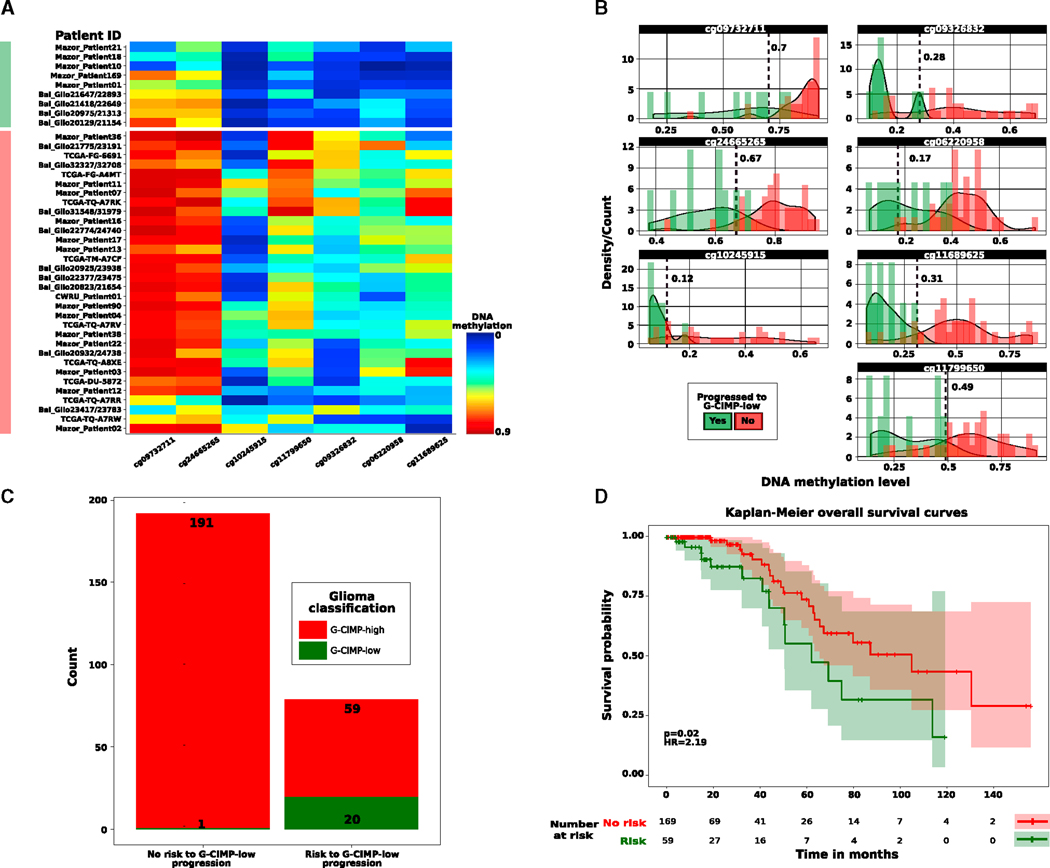
Clinical Application of Malignant Progression to G-CIMP-Low (A) Heatmap of DNA methylation data. Rows represent initially LGG G-CIMP-high tumors that progress to grade IV G-CIMP-low at first recurrence (labeled in green) and initially LGG G-CIMP-high tumors that retain their original G-CIMP-high phenotype at first recurrence with normal-like or indolent diseases (labeled in red). Glioma samples are sorted by hierarchical clustering. Columns represent the candidate predictive clinical biomarkers identified after supervised analysis of DNA methylation between the two tumor groups mentioned above sorted by hierarchical clustering (n = 7; unadjusted p < 0.05, absolute difference in mean methylation beta value > 0.2). The saturation of either color (scale from blue to red) reflects the magnitude of the difference in DNA methylation level. (B) Beta value thresholds that more specifically distinguish the primary glioma cases that progress to the aggressive G-CIMP-low phenotype from those primary glioma cases that relapse without malignant transformation and progression to the G-CIMP-low phenotype are represented and used to dichotomize the DNA methylation data in an independent validation cohort (n = 271). (C and D) Predictive clinical biomarkers of G-CIMP-low progression correlate with epigenomic subtype (C) and patient outcomes (D).

Our study demonstrates that G-CIMP-low tumor entities at first recurrence resemble *IDH*-wild-type GBMs known to exhibit an aggressive phenotype (Figures 2, 3, and 4). LGG relapse and malignant progression to GBM are highly variable and unpredictable by the 2016 WHO classification of diffuse gliomas (Sanai et al., 2011; Louis et al., 2016). To test whether G-CIMP-high to G-CIMP-low malignant transformation can be predicted from LGG G-CIMP-high primary diffuse gliomas, we performed a supervised analysis between DNA methylation of G-CIMP-high primary tumors progressing to the G-CIMP-low phenotype at first recurrence and G-CIMP-high primary tumors that retain their G-CIMP-high epigenetic profiling through glioma recurrence as a form of epigenetic memory (Wilcoxon rank-sum test, p < 0.05, absolute difference in mean methylation beta value > 0.2). We uncovered a set of candidate predictive biomarker signatures composed of 7 hypomethylated CpG sites in G-CIMP-high primary tumors that shifted their epigenomic profile and progressed to GBMs upon disease relapse (Figure 5A).

Next we sought to determine the usefulness of our biomarkers to predict gliomas at the time of initial surgical diagnosis at high risk for recurrence with a G-CIMP-low malignant phenotype. Toward this aim, we dichotomized the data using beta value thresholds that more specifically distinguished the primary glioma cases that relapse as progressed G-CIMP-low diseases from primary glioma cases that relapse as normal-like or indolent diseases. The beta value cutoff for each CpG probe was as follows: cg09732711 (0.7), cg09326832 (0.28), cg24665265 (0.67), cg06220958 (0.17), cg10245915 (0.12), cg11689625 (0.31), and cg11799650 (0.49) (Fisher’s exact test, FDR = 0.03; prognostication value and FDR were assigned at n ≥ 5 probes) (Figure 5B). We then investigated and validated the predictive value of these DNA methylation-based biomarkers in an independent cohort of 271 TCGA and non-TCGA primary gliomas previously classified in Ceccarelli et al. (2016) as IDH mutant non-Codel G-CIMP-high (n = 250) and IDH mutant non-Codel G-CIMP-low (n = 21). These 271 primary glioma samples were obtained from published datasets (Sturm et al., 2012; Turcan et al., 2012; Mur et al., 2013; Ceccarelli et al., 2016). We found that the possible clinical biomarker signatures identified here successfully predicted 29% of tumors (79 of 271) belonging to the “risk group,” including 95% (20 of 21) previously classified as G-CIMP-low primary tumors, with clinical relevance for patient overall survival (log rank p = 0.02, hazard ratio [HR] = 2.19) (Figures 5C and 5D; Table S5). These results provide insights into the tumorigenic events that contribute to G-CIMP progression, with opportunity for further targeted therapy exploitation as well as a inclusion in clinical trials design to impede or prevent tumor malignant transformation and progression to G-CIMP-low, an *IDH*-wild-type GBM-like tumor phenotype associated with *IDH* mutant non-Codel gliomas.

## DISCUSSION

The limited availability of clinical annotation and fresh tumor specimens representing transitional stages from tumor initiation to progression is an important barrier to effectively improving the therapeutic strategies and clinical outcomes for GBM patients. We describe the spatial and temporal epigenomic landscape of brain cancer evolution through comprehensive analysis of 200 longitudinal tumor biopsies derived from 77 glioma patients. To date, this represents the largest longitudinal adult diffuse glioma cohort (grade II to IV) with DNA methylation profiles spanning more than 450,000 CpGs to understand the epigenome- based evolution of gliomas.

*IDH*-wild-type and *IDH* mutant 1p-19q co-deleted glioma cases did not change dramatically in terms of their epigenomic profiles, but, among the IDH mutant non-Codel gliomas, we defined distinct patterns of epigenetic shifts throughout the course of tumor recurrence. Our large cohort of longitudinal CIMP gliomas allowed us to discover an intra-subtype heterogeneity relative to G-CIMP-high primary tumors with specific clinical outcomes further down the road of glioma evolution. Specifically, we observed a large subset of *IDH* mutant LGG G-CIMP-high tumor patients (37 of 53, 70%) that retained their normal-like epimethyl phenotype as a form of epigenetic memory when relapsed, but only a rare proportion of *IDH* mutant LGG G-CIMP-high tumor patients (9 of 53, 17%) underwent disease progression as G-CIMP-low epimethyl phenotype when relapsed for the first time. Identification of a subpopulation of G-CIMP-high tumors carrying the worst prognosis has crucial clinical implications for the assessment and therapeutic management of individual aggressive LGGs at risk for malignant recurrences and acquisition of an *IDH*-wild-type and stem cell-like glioblastoma phenotype that could not be predicted by histopathological grading at primary diagnosis. The discovery that a set of classical G-CIMP-high tumors at diagnosis are primed to recur toward a much more aggressive G-CIMP-low tumor phenotype prompted us to identify possible clinical biomarkers embedded in the primary tumors that could allow us to predict malignant evolution of G-CIMP methylomes. Remarkably, we uncovered 7 predictive biomarkers that identify, with high sensitivity and specificity, glioma patients at high risk for recurrence with a G-CIMP-low tumor. This information will allow neuro-oncologists to correctly predict, at the time of initial diagnosis, the evolution of the disease, identifying at-risk patients who may need more aggressive therapies. Such markers that define patient progression at primary diagnosis could potentially allow one to design in vitro and patient-derived xenograft models from these fresh tissues to study and evaluate the functional characterization and mechanisms by which G-CIMP-low evolves from G-CIMP-high. The finding also sheds light on the evolutionary trajectory of initial LGGs, suggesting that GBMs develop by different mechanistic epigenetic reprogramming pathways in response to different selective influences or microenvironmental injuries.

Our observation that *de novo* (primary) G-CIMP-low tumors share epigenome-wide features with acquired (recurrent) G-CIMP-low tumors provides the lay basis to support the notion that these tumors can be considered two separate tumor entities. Therefore, the establishment of a G-CIMP-low methylome in malignant recurrent gliomas reflects the response of transformed cells to the tumor microenvironment, which may involve the interaction of epigenetic selective pressure (possibly because of response to therapy) and immune, stromal, and vascular cells. Given the large sample size for this study, which allowed us to achieve statistical power, we sought to further our understanding of this malignant transformation by investigating the genomic DNA motif signature that is associated with this progression phenotype. In-depth motif analysis led to the identification of a STAT3 DNA signature at hypomethylated shores and intergenic open sea genomic sites in G-CIMP-low recurrent versus G-CIMP-low primary (fold enrichment = 4.36). STAT3 is known to play a role as a master regulator of mesenchymal differentiation in glioma cells (Carro et al., 2010), and, hence, this provides meaningful insights into the evolution of G-CIMP-low recurrent cells along the aberrant mesenchymal lineage transformation and unfavorable patient outcomes because these tumors can emerge as secondary GBMs. This hypothesis is also supported by the higher mDNAsi in G-CIMP-low recurrent versus the precursor G-CIMP-high counterparts. Additionally, G-CIMP-low recurrent tumors can be distinguished from their parental G-CIMP-high counterparts by acquisition of DNA demethylation abnormalities at intergenic enhancers associated with the c-JUN/AP-1 binding motif, which were strongly reflective of (epi)genomic and stemness signatures of *IDH*-wild-type primary GBMs. Interestingly, a recent study demonstrated that c-JUN N-terminal phosphorylation regulates the *DNMT1* gene promoter, leading to DNA hypermethylation that is similar to the G-CIMP phenotype in LGGs and proneural GBMs and correlates with downregulation of mesenchyme-related genes and reduced cell migration and invasiveness (Heiland et al., 2017). Altogether, the aforementioned findings would imply that DNA methylation loss associated with G-CIMP-low recurrence reflects chromatin remodeling events orchestrated by the interrelationship between the tumor microenvironment and the TFs c-JUN/AP-1 and STAT3. In our study, we observed 100% of G-CIMP-low at recurrence as grade IV tumors; however, not all grade IV gliomas resembled G-CIMP-low, suggesting that grade may not be the only determinant of G-CIMP-low cell identity in this rare subset of aggressive *IDH* mutant 1p-19q non-Codel gliomas.

Evidence is emerging that epigenetic abnormalities recapitulate somatic mutation events on cell cycle networks throughout relapse and malignant progression of LGG G-CIMP glioma cells to GBMs (Mazor et al., 2015). Interestingly, we are reporting the existence of a small set of tumor specimens within the *IDH* mutant LGG G-CIMP-high primary subtype exhibiting a G-CIMP-intermediate epimethyl pattern when relapsed for the first time (7 of 53, 13%). G-CIMP-intermediate reflects epigenomic signatures of stemness comparable with G-CIMP-low recurrent. Our whole-genome rearrangement results (Figures S4A–S4D) provide evidence that intra-subtype heterogeneity relative to G-CIMP-high primary tumors is associated with a higher frequency of loss of the cell cycle genes *CDKN2A* and *CDKN1B* found in G-CIMP-intermediate recurrent tumors. Loss of the cell cycle inhibitor protein CDKN1B is a positive regulator of self-renewal and pluripotency in human embryonic stem cells (Menchón et al., 2011). Although IDH mutation initiates gliomagenesis and is retained upon recurrence, a recent work suggested that neither mutant *IDH1* nor the oncometabolite 2HG are required for glioma recurrence. Moreover, recurrent glioma cells can delete or amplify the IDH1 mutant or wild-type allele, which is followed by clonal expansion and recurrence of tumors that resembled the G-CIMP-low primary subtype. This raises the possibility that *IDH1* copy number alterations (CNAs) contribute to altering the G-CIMP-low tumor methylome (Mazor et al., 2017). In conjunction, these findings provide evidence to mechanistically hypothesize that G-CIMP-intermediate at recurrence recapitulates an early stage of chromatin remodeling downstream of *IDH1* CNAs and genomic abnormalities on the tumor suppressor genes *CDKN2A* and *CDKN1B* to evade cell cycle control at G1. This would favor phenotype switching and confer tumor undifferentiation (stem cell-like phenotype) and a selective subclonal oncogenic growth advantage toward G-CIMP-low malignant recurrent cells. This hypothesis has potential implications to abrogate the establishment and progression of a malignant glioma recurrent phenotype, suggesting possible synergistic activity of an *IDH* mutant inhibitor (to target a phenotypic subpopulation of G-CIMP tumor cells with a tumorigenic advantage) combined with targeted therapy aimed at re-establishing the tumor suppressor gene function at *CDKN2A* and *CDKN1B* gene loci (to target a phenotypic subpopulation of G-CIMP tumor cells with tumor-propagating and tumor relapse advantages). Therefore, eradication of cells showing an early-stage transition relative to G-CIMP progression may be an appealing strategy that should be exploited to control malignant gliomas. The spatial and temporal dynamics of G-CIMP epimethyl patterns identified in our current study (G-CIMP-high to G-CIMP-high and G-CIMP- high to G-CIMP-low) allowed us to estimate the effect of somatic mutations alongside the evolution of G-CIMP methylomes in the exomes of initially LGG G-CIMP-high patients whose tumors relapsed as G-CIMP-high (n = 8) or G-CIMP-low (n = 4). We showed that G-CIMP-low recurrent tumors had the highest total number of somatic mutations in relation to their G-CIMP-high primary counterparts and G-CIMP-high at first recurrence (Figure S4E). This finding suggests that genomic instability acquired by G-CIMP-low-progressed tumors accumulates downstream of epigenetic reprogramming. Furthermore, this provides another layer of evidence that genetic/epigenetic divergence exists in the G-CIMP-high subtype at primary disease. Johnson et al. (2014) reported that the 4 patients identified in our current study as progressing to G-CIMP-low at recurrence (Figures 2 and S4E) harbored a signature of temozolomide (TMZ)-induced mutagenesis in the RB and AKT-mTOR pathways, following an alternative evolutionary path to GBM. Despite the fact that driver mutations in these pathways and the hypermutator phenotype can emerge at disease relapse after chemotherapy with TMZ (Johnson et al., 2014), we identified convergent genetic alterations in G-CIMP-low primary tumors (Ceccarelli et al., 2016; Figure S5). This reinforces the idea that distinct oncogenic selective pressures would drive the evolution of G-CIMP-low primary tumors (cell-intrinsic injury?) and G-CIMP-low recurrent tumors (epigenetic plasticity as an adaptation to external cellular stimuli driven by therapy?).

Although recent reports have highlighted pronounced epigenetic differences between LGG primary gliomas and GBM recurrent gliomas, these studies have grouped tumors by either grade or genomic alterations. In our study, we took a more holistic approach guided by our recent findings that the 2016 WHO classification of diffuse gliomas can be further divided by epigenomic subtypes that are prognostically advantageous over both *IDH* mutation status and histopathological grade. Collectively, our data provide a conceptual framework to explore the molecular drivers of genetic alterations and epigenetic plasticity contributing to G-CIMP malignant evolution toward an *IDH*-wild-type and mesenchymal/stem cell-like glioblastoma phenotype, a platform for identifying tumors and patients that best respond to certain therapies, and predictive biomarkers for refining clinical trial designs to determine optimal management of patients at risk for malignant glioma recurrences.

## EXPERIMENTAL PROCEDURES

Further details and an outline of the resources used in this work can be found in the Supplemental Experimental Procedures.

### Patient and Sample Characteristics

Specimens were obtained from patients with appropriate consent from institutional review boards. Details of sample preparation are described in the Supplemental Experimental Procedures. Sample IDs and tissue source sites from our entire longitudinal glioma cohort are listed in Table S1.

### Data and Software Availability

Data visualization and statistical analysis were performed using R software packages (https://www.r-project.org). The raw 450,000 DNA methylation data reported in this paper has been deposited to Mendeley Data at https://data.mendeley.com/datasets/hx566mwxnm/. All other raw data are available through Genomics Data Commons (in the case of TCGA, the data are accessible via TCGAbiolinks; Colaprico et al., 2016) or have been described in previous studies (Mazor et al., 2015; Bai et al., 2016; Mazor et al., 2017). For level 1 TCGA/GDC ‘‘Illumina HumanMethylation450’’ data acquisition (version 12 for LGG and version 6 for GBM), we used the Bioconductor package TCGAbiolinks version 1.1.12 (Colaprico et al., 2016). In addition to TCGA data, we obtained a published dataset of 81 (Mazor et al., 2015, 2017) and a dataset of 48 (Bai et al., 2016) longitudinally collected gliomas (a complete list of samples and their respective IDs are available in Table S1). Probe-level signals for individual CpG sites (raw IDAT files) were subjected to background correction, global dye bias normalization, calculation of the DNA methylation level, and detection p values (Triche et al., 2013) using the Bioconductor package methylumi version 2.16.0. Longitudinal glioma samples were classified as either *IDH*-wild-type (classic-like, mesenchymal-like, LGm6-GBM, and PA-like) or *IDH* mutant (Codel, G-CIMP-high, and G-CIMP-low) DNA methylation subtypes using the CpG methylation signatures previously defined by our group (Ceccarelli et al., 2016; tcga-data.nci.nih.gov/docs/publications/lgggbm_2016/PanGlioma_MethylationSignatures.xlsx) and the R package caret version 6.0–76 and randomForest version 4.6–12. RF probability indices are provided in Table S1. We used the Wilcoxon rank-sum test followed by multiple testing using the Benjamini and Hochberg (BH) method for FDR estimation (Benjamini and Hochberg, 1995) to identify differentially methylated sites between two groups of study. *De novo* and known motif discovery analyses were conducted using Hypergeometric Optimization of Motif Enrichment (HOMER) version 4.9 with the perl script findMotifGenome.pl (Heinz et al., 2010). Raw outputs from HOMER reported in this paper can be found at Mendeley Data at https://data.mendeley.com/datasets/hx566mwxnm/.

## Supplementary Material

1

2

3

4

5

6

## Figures and Tables

**Table 1.** Clinical Characteristics of the Glioma Primary Cohort with Known IDH1/1p-19q Status

Primary Glioma (n = 74) Feature	IDH-mut Non-Codel (n = 54)	IDH-mut Codel (n = 7)	IDH-WT (n = 13)

Clinical			

Cohort (n, %)			

TCGA	13(24.07%)	3 (42.86%)	13(100%)
Non-TCGA	41 (75.93%)	4(57.14%)	0 (0%)

2016 WHO (n, %)			

Oligodendroglioma grade II	0 (0%)	4(57.14%)	0 (0%)
Astrocytoma grade II	47 (87.04%)	0 (0%)	0 (0%)
Anaplastic oligodendroglioma grade III	0 (0%)	3 (42.86%)	2(15.38%)
Anaplastic astrocytoma grade III	6(11.11%)	0 (0%)	0 (0%)
Glioblastoma grade IV	1 (1.85%)	0 (0%)	11 (84.62%)

Gender (n, %)			

Female	22 (40.74%)	3 (42.86%)	5 (38.46%)
Male	32 (59.26%)	4(57.14%)	8 (61.54%)

Age (n, %)			

≤ 40 years	41 (75.93%)	2 (28.57%)	3 (23.08%)
> 40 years	13(24.07%)	5 (71.43%)	10(76.92%)

Treatment			

Radiation after Surgery (n, %)			

Yes	18(33.33%)	3 (42.86%)	13(100%)
No	36 (66.67%)	4(57.14%)	0 (0%)

Adjuvant TMZ			

Yes	11 (20.37%)	1 (14.29%)	6(46.15%)
No	40 (74.07%)	6(85.71%)	5 (38.46%)
Unknown	3 (5.56%)	0 (0%)	2(15.38%)

Percentages were calculated as a proportion of a total amount of tumor samples in the glioma primary cohort with known IDH1/1p-19q status by group. In cases where more than one tumor fragment per primary surgery were investigated, each case was counted once to avoid overrepresentation of data.

**Table 2.** Clinical Characteristics of the Glioma First Recurrent Cohort with Known IDH1/1p-19q Status

First recurrent Gliomas (n = 69) Feature	IDH-mut Non-Codel (n = 50)	IDH-mut Codel (n = 6)	IDH-WT (n = 13)

Clinical			

Cohort (n, %)			

TCGA	10(20%)	3 (50%)	13 (100%)
Non-TCGA	40 (80%)	3 (50%)	0 (0%)

2016 WHO (n, %)			

Astrocytoma grade II	13(26%)	0 (0%)	0 (0%)
Anaplastic oligodendroglioma grade III	0 (0%)	6(100%)	0 (0%)
Anaplastic astrocytoma grade III	18(36%)	0 (0%)	1 (7.69%)
Glioblastoma grade IV	19(38%)	0 (0%)	12 (92.31%)

Gender (n, %)			

Female	21 (42%)	3 (50%)	5 (38.46%)
Male	29 (58%)	3 (50%)	8(61.54%)

Age (n, %)			

≤ 40 years	33 (66%)	0 (0%)	3 (23.08%)
> 40 years	17(34%)	6(100%)	10 (76.92%)

Treatment			

Radiation after Surgery (n, %)			

Yes	6(12%)	3 (50%)	1 (7.69%)
No	8(16%)	0 (0%)	1 (7.69%)
Unknown	36 (72%)	3 (50%)	11 (84.62%)

Adjuvant TMZ			

Yes	5(10%)	3 (50%)	0 (0%)
No	6(12%)	0 (0%)	0 (0%)
Unknown	39 (78%)	3 (50%)	13(100%)

Percentages were calculated as a proportion of a total amount of tumor samples in the glioma first recurrent cohort with known IDH1/1p-19q status by group. In cases where more than one tumor fragment per first recurrent surgery were investigated, each case was counted once to avoid overrepresentation of data.

## Highlights

- Intra-subtype heterogeneity of initially G-CIMP-high carries worst prognosis
- G-CIMP-low is defined by DNA signature motifs for STAT3 and c-JUN/AP-1 at recurrence
- G-CIMP-low at recurrence mimics an IDH-wild-type and stem cell-like primary GBM
- Predictive biomarkers of glioma malignant transformation and recurrence are observed at diagnosis
