# Supplementary material for: A Distinct DNA Methylation Shift in a Subset of Glioma CpG Island Methylator Phenotypes during Tumor Recurrence
Source: Cell Rep. Author manuscript; Available in PMC 2022 Feb 21. (PMC8859991; doi:10.1016/j.celrep.2018.03.107)
Supplement: 1 [file NIHMS1777196-supplement-1.pdf]

Cell Reports, Volume *23*

Supplemental Information

A Distinct DNA Methylation Shift in a Subset of Glioma CpG Island Methylator Phenotypes during Tumor Recurrence

Camila Ferreira de Souza, Thais S. Sabedot, Tathiane M. Malta, Lindsay Stetson, Olena Morozova, Artem Sokolov, Peter W. Laird, Maciej Wiznerowicz, Antonio Iavarone, James Snyder, Ana deCarvalho, Zachary Sanborn, Kerrie L. McDonald, William A. Friedman, Daniela Tirapelli, Laila Poisson, Tom Mikkelsen, Carlos G. Carlotti Jr., Steven Kalkanis, Jean Zenklusen, Sofie R. Salama, Jill S. Barnholtz-Sloan, and Houtan Noushmehr

SUPPLEMENTAL EXPERIMENTAL PROCEDURES

CONTACT FOR REAGENT AND RESOURCE SHARING

Further information and requests for resources and reagents should be directed to and will be fulfilled by the Lead Contact Houtan Noushmehr (hnoushm1@hfhs.org).

EXPERIMENTAL MODEL AND SUBJECT DETAILS

TCGA/GDC (https://portal.gdc.cancer.gov), Sturm et al. (2012), Turcan et al. (2012), Guintivano et al. (2013), Mur et al. (2013), Mazor et al. (2015), Bai et al. (2016), and Mazor et al. (2017). Molecular data from our own cohort collected from patients diagnosed with primary and recurrent gliomas undergoing surgical resection were snap frozen in liquid nitrogen and subjected to pathology quality control metrics, as previously described (Cancer Genome Atlas Research Network, 2008). Specimens were obtained from patients with appropriate consent. Sample IDs and tissue source sites from our entire longitudinal glioma cohort are listed in Table S1.

METHOD DETAILS

Molecular and clinical covariates

Molecular data elements comprise *IDH1* and 1p-19q status. All TCGA/GDC cases were manually followed up by the individual tissue source sites and represents a complete update of new cases as well as cases from the previous publications (Brennan et al., 2013); (Ceccarelli et al., 2016). Clinical covariates comprise grade, histology, gender, age at primary and recurrent tumor diagnoses, radiation therapy, chemotherapy based on Temozolomide (TMZ), overall survival, and survival since first recurrence. Survival since first recurrence was defined as follows: (patient overall survival) - (time elapsed between primary and first recurrent tumor surgeries) (Table S1). Clinical data were updated as of September 2015 by contacting the individual tissue source sites, in case of TCGA/GDC, and as of January 2016 and April 2016 by previously published reports in case of (Bai et al., 2016; Mazor et al., 2015, 2017). Survival curves were estimated using the Kaplan-Meier method and Cox proportional hazards analysis.

Illumina human methylation 450K array

Primary and recurrent samples from CWRU_Patient01, CWRU_Patient02, and CWRU_Patient03 were obtained from Case Western Reserve University. Recurrent samples from previously deposited primary matched TCGA samples (i.e. TCGA-TQ-A7RR, TCGA-TQ-A7RW and TCGA-FG-6691) were also included in this study to expand the total number of available glioma primary and recurrent pairs. In the case of the TCGA GBM recurrent cohort (n=12), the initial primary DNA was profiled using the GoldenGate I and II platforms (~6,000 CpGs) (Noushmehr et al., 2010); (Brennan et al., 2013) while the matched recurrent DNA was profiled using the Infinium HumanMethylation450 bead arrays platform (also commonly referred to as 450K). In order to effectively analyze the epigenomics across all available glioma recurrent samples for this study, we generated 450K Illumina DNA methylation for these primary TCGA GBMs (n=12), approved by the TCGA director, Dr. Jean Zenklusen (Table S2). Genomic DNA from the gliomas (n=24 samples) was extracted and purified using AllPrep® DNA/RNA/miRNA Universal kit (QIAGEN), following the manufacturer's instructions. Only DNA samples with an absorbance A260/A280 nm ratio ≥1.8 were used for microarray hybridization. Sodium bisulfite conversion of the genomic DNA was done with EZ DNA methylation Kit (ZymoResearch, USA) following the manufacturer's guidelines. Briefly, purified genomic DNA (1µg) was bisulfite converted and processed on Infinium HumanMethylation450 bead arrays (Illumina Inc.), as described previously (Ceccarelli et al., 2016). Raw 450K DNA methylation data (samples generated for the purpose of this study) is available through Mendeley Data (accession ID: https://data.mendeley.com/datasets/hx566mwxnm/). All other raw data are available through Genomics Data Commons (in the case of TCGA and the data is accessible via TCGAbiolinks (Colaprico et al., 2016) or described in previous studies (Mazor et al., 2015); (Bai et al., 2016; Mazor et al., 2017).

Regulatory genomics interrogated by 450K

The genomic location of 450K probes was divided into those within CpG islands (CGIs), in shores, or in open seas (with and without overlapping gene bodies) using UCSC genome table browser (hg19) CGI genomic annotation. UCSC adopts the following criteria to define CGI: GC content of at least 50%, length greater than 200 bp, and ratio of observed number of CG dinucleotides to the expected number of G and C in the DNA segment greater than 0.6 (genome.ucsc.edu). We annotated shores as genomic regions of 2,000 bp upstream and downstream flanking CGI boundaries, and open seas as genomic regions located neither in CGIs nor in shores. Accordingly, 31% of 450K probes are located within CGIs, 21% in shores, and 48% in open seas. 132,363 450K probes show overlapping with candidate enhancers that were previously described by (Yao et al., 2015). Briefly, these functional

genomic elements are distal 2,000 bp away from transcription start sites (TSSs), DNA hypomethylated in tumors when compared with normal controls, correlated to cancer-specific transcription factors, and overlap three enhancer databases: 1) REMC (Roadmap Epigenomics Mapping Consortium) plus 2) ENCODE (chromHMM for 98 tissues or cell lines), and 3) FANTOM5 (enhancers defined by eRNAs for 400 distinct cell types (Yao et al., 2015). An annotation of 450K bivalent chromatin domains in human embryonic stem cells was obtained from a published dataset. Accordingly, bivalent probes are mostly located in CGI-rich promoters and possess molecular features associated with depletion of retroelements (LINE, SINE, and LTR) in the genomic region directly bordering the TSSs, enrichment of H3K4me3 and H3K27me3 histone modifications (minimum overlapping size of 1 Kb), and occupancy by PRC2 and PolII complexes (Court and Arnaud, 2017).

Computational methods

Illumina 450K array preprocessing

For level 1 TCGA/GDC "Illumina HumanMethylation450" data acquisition (version 12 for LGG and version 6 for GBM) we used the Bioconductor package TCGAbiolinks version 1.1.12 (Colaprico et al., 2016). A total of 59 matched primary and recurrent gliomas, including 35 LGG and 24 GBM samples were profiled through a 450K array platform which interrogates DNA methylation levels of 485,577 human methylation sites. In addition to the TCGA data, we obtained a published dataset of 81 (Mazor et al., 2015, 2017) and a dataset of 48 (Bai et al., 2016) longitudinally collected gliomas (complete list of samples and their respective IDs are available in Table S1). Probe-level signals for individual CpG sites (raw IDAT files) were subjected to background correction, global dye-bias normalization, calculation of DNA methylation level, and detection p-values (Triche et al., 2013) using the Bioconductor package methylumi version 2.16.0. The DNA methylation level for each locus is measured as a beta-value score ($\beta = (M/(M+U))$ in which M and U represent the mean methylated and the mean unmethylated signal intensities for each *locus*, respectively. Beta-values score range from zero to one with scores of zero indicating complete unmethylated DNA and scores of one indicating complete methylated DNA. A p-value detection of each data point is used to compare the signal intensity difference between the analytical CpG sites and a set of negative control CpG sites represented in the array. A p-value of greater than 0.05 is not statistically significantly different from the background and is masked as "NA". Probes that are designed for sequences with known germline polymorphisms (Illumina supplementary SNP list version 2, downloaded 12 December, 2016) and the X and Y chromosomes were filtered out before supervised analysis (466,835 probes remained following filtering).

Classification of longitudinal gliomas

Longitudinal glioma samples were classified as either IDH-wildtype (Classic-like, Mesenchymal-like, LGm6-GBM, and PA-like) or IDH-mutant (Codel, G-CIMP-high, and G-CIMP-low) DNA methylation subtypes using the CpG methylation signatures previously defined by our group (Ceccarelli et al., 2016) (tcga-data.nci.nih.gov/docs/publications/lgggbm_2016/PanGlioma_MethylationSignatures.xlsx). We evaluated the performance of the Random Forest (RF) machine learning prediction model by training on a random set of either 80% of 430 IDH-wildtype samples or 80% of 448 IDH-mutant adult diffuse primary LGG-GBM TCGA samples (tcga-data.nci.nih.gov/docs/publications/lgggbm_2016/LGG.GBM.meth.txt), and then evaluated the performance on the remaining 20% of glioma samples. Given the high specificity and sensitivity of our model (accuracy > 95% on average), we tested the prediction model on the primary and recurrent gliomas (n=200 tumor fragments) to classify them according to the seven DNA methylation glioma subtypes using the RF approach and the R packages caret version 6.0-76 and randomForest version 4.6-12. RF probability indices are provided in Table S1.

QUANTIFICATION AND STATISTICAL ANALYSIS

Supervised analysis of DNA methylation

We used the Wilcoxon rank-sum test followed by multiple testing using the Benjamini & Hochberg (BH) method for false discovery rate (FDR) estimation (Benjamini and Hochberg, 1995) to identify differentially methylated sites between two groups of study. The 84 CpG probes (Table S3) were defined by comparing a rare set of *de novo* (primary) G-CIMP-low tumors (n=12) and acquired (first recurrent) G-CIMP-low tumors (n=9), using the following criteria: FDR < 0.05 (adjusted *p*) and absolute difference in mean methylation beta-value > 0.2. The 712 probes (Table S4) were defined by comparing a core set of 9 cases that underwent malignant transformation from G-CIMP-high at primary diagnosis to G-CIMP-low at first recurrence, using the following criteria: FDR < 0.05 (adjusted *p*) and difference in mean methylation beta-value < -0.4 and > 0.5. The 7 probes that define prediction were uncovered by comparing a core set of 9 G-CIMP-high primary tumors progressing to the G-CIMP-low phenotype and 32 G-CIMP-high primary tumors that retained the G-CIMP-high epigenetic profiling through glioma

recurrence. We used the following criteria: $p < 0.05$ (unadjusted) and absolute difference in mean methylation beta value > 0.2.

Motif discovery

De novo and known motif discovery analyses were conducted using Hypergeometric Optimization of Motif EnRichment (HOMER) version 4.9 (Heinz et al., 2010). The perl script findMotifGenome.pl was used to search for conserved DNA binding sequences (8-20 bp motifs) associated with genomic differentially methylated regions (DMRs) (adjusted $p < 0.05$), using the following criteria: hg19 genome assembly, 200 bp upstream and downstream flanking each target CpG site, and expected genome-wide distribution of 450K probes as background. Raw outputs from HOMER can be found at Mendeley Data (accession ID: https://data.mendeley.com/datasets/hx566mwxnm/).

Chromatin state (chromHMM) analysis

ChromHMM data, which define 18 distinct chromatin states by combining 6 histone markers (i.e. H3K27ac, H3K4me1, H3K4me3, H3K36me3, H3K27me3, and H3K9me3) across 98 reference human epigenomes were downloaded from the NIH Roadmap Epigenomics Consortium (Roadmap Epigenomics Consortium et al., 2015). Data shown in Figure S3 reflect the chromatin states in somatic cells and stem cells for the genomic regions that map to the 28 probes defined as differentially hypermethylated and 684 probes defined as differentially hypomethylated in G-CIMP-low recurrent cases in relation to their G-CIMP-high primary counterparts.

Fusion transcript and DNA rearrangement analyses

We investigated fusion transcript events and DNA rearrangements in a total of 30 matched primary and recurrent TCGA LGG samples derived from 13 patients with available RNA sequencing and whole-genome sequencing data for the primary tumor and at least one recurrent sample. RNA sequencing reads were analyzed using deFuse package version 0.6.0 (McPherson et al., 2011). Candidate fusions were filtered based on the following deFuse parameters:

- Splitr_count ≥ 5 (5 or more split reads supporting the fusion)
- Span_count ≥ 10 (10 or more spanning reads supporting the fusion)
- Read_through ~ "N" (fusion is not a readthrough)
- Adjacent ~ "N" (fusion does not involve adjacent genes)
- Altsplice ~ "N" (fusion can not be explained by alternative splicing)
- Min_map_count=1 (at least one spanning read supporting the fusion is uniquely mapped)
- ORF ~ "Y" (fusion preserves the open reading frame)

The deFuse fusion predictions were manually reviewed using blat analysis (Kent, 2002) of the breakpoint sequence in the UCSC Genome Browser (Kent et al., 2002). Whole-genome DNA rearrangements were identified using the BamBam tool (Sanborn et al., 2013), following the standard recommendation posted on the Five3 Genomics website (dna.five3genomics.com). Briefly, variants with at least 6 reads with an average mapping quality > 30 were ranked as follows: +2 if read support > 15; +4 if split reads are found; +5 if ORF is preserved in a fusion gene; +4 if "Near fusion" (correct orientation but improper phase); +2 if deletion could cause a loss of any part of a gene; +1 if variant interrupts a gene. deFuse and BamBam predictions were combined to define the genomic events identified by both methods. Genomic rearrangements for each of the 13 patients with primary and recurrent tumor samples is plotted as circos plots (Figures S4A-S4D). A summary of manually curated DNA and RNA rearrangements for each patient is available at Mendeley data (https://data.mendeley.com/datasets/hx566mwxnm/).

Statistics

Data visualization and statistical analysis were performed using R software packages (www.r-project.org) and Bioconductor (Gentleman et al., 2004).

DATA AND SOFTWARE AVAILABILITY

The raw 450K DNA methylation data generated in this study have been deposited at Mendeley Data (accession ID: https://data.mendeley.com/datasets/hx566mwxnm/)

Supplemental Files available at mendeley data:

Rearrangements.xls - A summary of DNA and RNA rearrangements organized by TCGA/GDC patient. Related to Figures S4A-D. This data have been deposited at Mendeley Data (accession ID: https://data.mendeley.com/datasets/hx566mwxnm/).

HOMER.zip - HOMER motif scans for the 84 and 712 differentially methylated CpG probes associated with malignant recurrence to G-CIMP-low. Related to Figures 3 and 4. This data have been deposited at Mendeley Data

(accession ID: https://data.mendeley.com/datasets/hx566mwxnm/).

RESOURCE TABLE

REAGENT or RESOURCE	SOURCE	IDENTIFIER
**Software and Algorithms**		
R 3.3.1	R Core Team, 2017	https://www.R-project.org
ggplot2 (v2.2.1)	Wickham, 2009	https://CRAN.R-project.org/package=ggplot2
TCGAbiolinks (v1.1.12)	Colaprico et al., 2016	http://bioconductor.org/packages/TCGAbiolinks/
Workflow to reproduce the stemness index	Malta et al., 2018	https://bioinformaticsfmrp.github.io/PanCanStem_Web/
Methylumi (v2.16.0)	Davis et al., 2015	http://bioconductor.org/packages/methylumi/
deFuse	McPherson et al., 2011	http://shahlab.ca/projects/defuse/
HOMER (v4.9)	Heinz et al., 2010	http://homer.ucsd.edu/homer/ngs/peakMotifs.html
**Deposited Data**		
Raw 450K DNA methylation data	This paper	https://data.mendeley.com/datasets/hx566mwxnm/
Raw outputs from HOMER	This paper	https://data.mendeley.com/datasets/hx566mwxnm/
DNA and RNA rearrangements	This paper	https://data.mendeley.com/datasets/hx566mwxnm/
TCGA glioma cohort	Ceccarelli et al., 2016	https://tcga-data.nci.nih.gov/docs/publications/lgggbm_2016/
Longitudinal glioma cohort	Mazor et al., 2015	EGAS00001001255
Longitudinal glioma cohort	Mazor et al., 2017	EGAS00001001854
Longitudinal glioma cohort	Bai et al., 2016	EGAS00001001588
Glioma validation set	Sturm et al., 2012	GEO: GSE36278
Glioma validation set	Turcan et al., 2012	GEO: GSE30339
Glioma validation set	Mur et al., 2013	GEO: GSE61160
Normal neuron and normal glial	Guintivano et al., 2013	GEO: GSE41826

cells datasets		
Stemness indices	Malta et al., 2018	https://gdc.cancer.gov/about-data/publications/PanCanStemness-2018
Chromatin State (ChromHMM)	(Roadmap Epigenomics Consortium et al., 2015)	http://www.roadmapepigenomics.org
FANTOM5	Yao et al., 2015	http://fantom.gsc.riken.jp/5/

SUPPLEMENTAL REFERENCES

Benjamini, Y., and Hochberg, Y. (1995). Controlling the False Discovery Rate - a Practical and Powerful Approach to Multiple Testing. J Roy Stat Soc B Met *57*, 289-300.

Colaprico, A., Silva, T.C., Olsen, C., Garofano, L., Cava, C., Garolini, D., Sabedot, T.S., Malta, T.M., Pagnotta, S.M., Castiglioni, I., et al. (2016). TCGAbiolinks: an R/Bioconductor package for integrative analysis of TCGA data. Nucleic Acids Res *44*, e71.

Court, F., and Arnaud, P. (2017). An annotated list of bivalent chromatin regions in human ES cells: a new tool for cancer epigenetic research. Oncotarget *8*, 4110–4124.

Gentleman, R.C., Carey, V.J., Bates, D.M., Bolstad, B., Dettling, M., Dudoit, S., Ellis, B., Gautier, L., Ge, Y., Gentry, J., et al. (2004). Bioconductor: open software development for computational biology and bioinformatics. Genome Biol *5*, R80.

Guintivano, J., Aryee, M.J., and Kaminsky, Z.A. (2013). A cell epigenotype specific model for the correction of brain cellular heterogeneity bias and its application to age, brain region and major depression. Epigenetics *8*, 290–302.

Heinz, S., Benner, C., Spann, N., Bertolino, E., Lin, Y.C., Laslo, P., Cheng, J.X., Murre, C., Singh, H., and Glass, C.K. (2010). Simple combinations of lineage-determining transcription factors prime cis-regulatory elements required for macrophage and B cell identities. Mol Cell *38*, 576–589.

Kent, W.J. (2002). BLAT --the BLAST-like alignment tool. Genome Res *12*, 656-664.

Kent, W.J., Sugnet, C.W., Furey, T.S., Roskin, K.M., Pringle, T.H., Zahler, A.M., and Haussler, D. (2002). The human genome browser at UCSC. Genome Res *12*, 996–1006.

McPherson, A., Hormozdiari, F., Zayed, A., Giuliany, R., Ha, G., Sun, M.G.F., Griffith, M., Heravi Moussavi, A., Senz, J., Melnyk, N., et al. (2011). deFuse: an algorithm for gene fusion discovery in tumor RNA-Seq data. PLoS Comput Biol *7*, e1001138.

Sanborn, J.Z., Salama, S.R., Grifford, M., Brennan, C.W., Mikkelsen, T., Jhanwar, S., Katzman, S., Chin, L., and Haussler, D. (2013). Double minute chromosomes in glioblastoma multiforme are revealed by precise reconstruction of oncogenic amplicons. Cancer Res *73*, 6036–6045.

Triche, T.J., Weisenberger, D.J., Van Den Berg, D., Laird, P.W., and Siegmund, K.D. (2013). Low-level processing of Illumina Infinium DNA Methylation BeadArrays. Nucleic Acids Res *41*, e90.

Yao, L., Shen, H., Laird, P.W., Farnham, P.J., and Berman, B.P. (2015). Inferring regulatory element landscapes and transcription factor networks from cancer methylomes. Genome Biol *16*, 105.

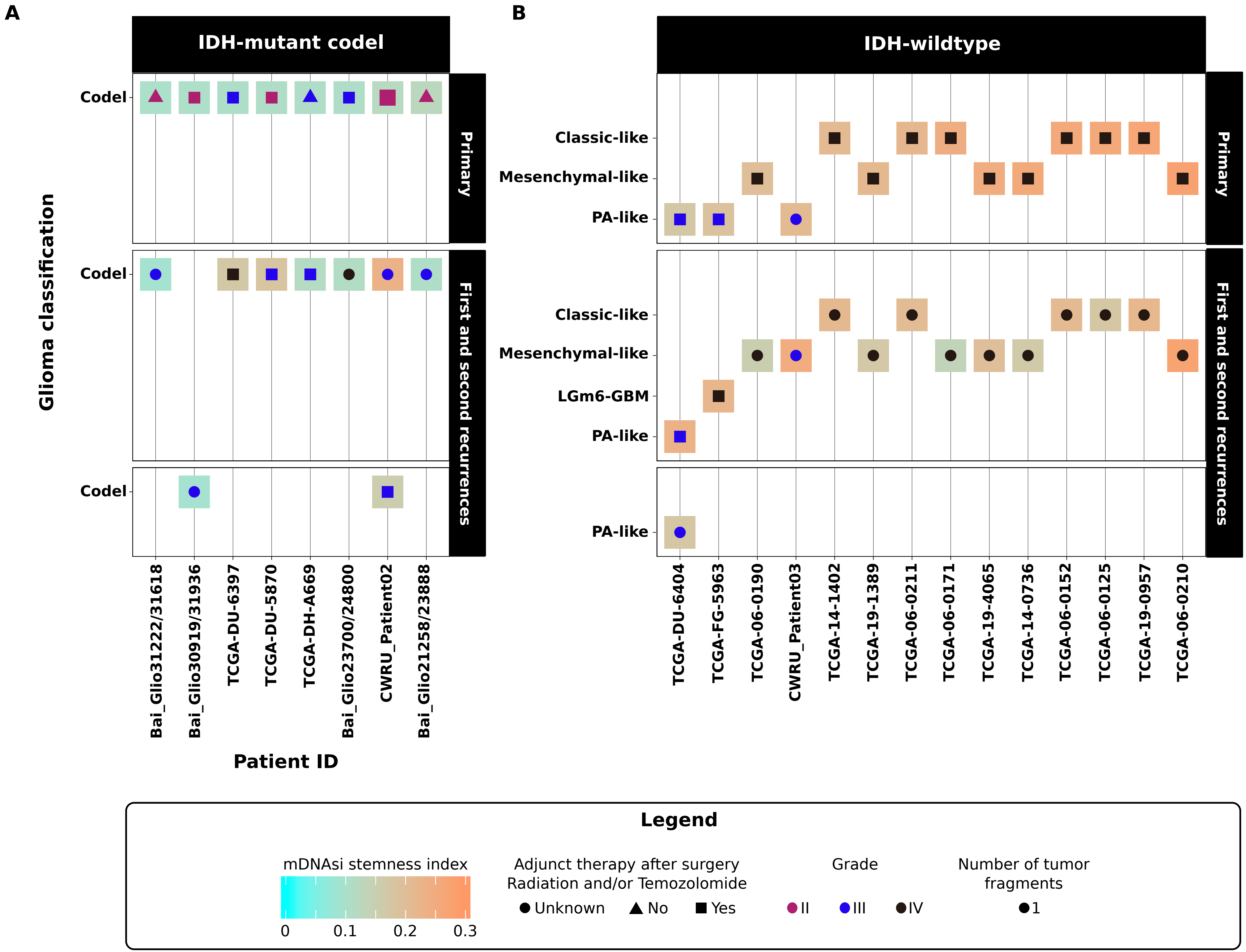
A
B
IDH-mutant codel
IDH-wildtype
Codel
Classic-like
Mesenchymal-like
PA-like
LGm6-GBM
Primary
First and second recurrences
Glioma classification
Patient ID
Bai_Glio31222/31618
Bai_Glio30919/31936
TCGA-DU-6397
TCGA-DU-5870
TCGA-DH-A669
Bai_Glio23700/24800
CWRU_Patient02
Bai_Glio21258/23888
TCGA-DU-6404
TCGA-FG-5963
TCGA-06-0190
CWRU_Patient03
TCGA-14-1402
TCGA-19-1389
TCGA-06-0211
TCGA-06-0171
TCGA-19-4065
TCGA-14-0736
TCGA-06-0152
TCGA-06-0125
TCGA-19-0957
TCGA-06-0210
Legend
mDNAsi stemness index
0
0.1
0.2
0.3
Adjunct therapy after surgery
Radiation and/or Temozolomide
Unknown
No
Yes
Grade
II
III
IV
Number of tumor
fragments
1

Figure S1. Temporal epigenomic phenotype of adult diffuse longitudinal gliomas harboring IDH-mutant codel and IDH-wildtype genotypes. Related to Figure 2. Schematic representation of matched primary and recurrent gliomas carrying (A) IDH-mutant 1p-19q co-deletion and (B) IDH-wildtype genotypes. Such glioma cases do not change dramatically in terms of their DNA methylation patterns from primary to recurrent diseases. Tumors are categorized according to their stem cell-like prevalence/degree of undifferentiation (stemness) by using the DNA methylation-based stemness index (mDNAsi) (a score value from 0 to 1) as a metric. Each box represents a patient tumor colored according to its mDNAsi at primary and recurrent stages of the disease. A symbol color, size, and shape within each box represents tumor grade, number of tumor fragments, and adjunct therapy (radiation and/or Temozolomide) received after surgery of primary and recurrent tumors.

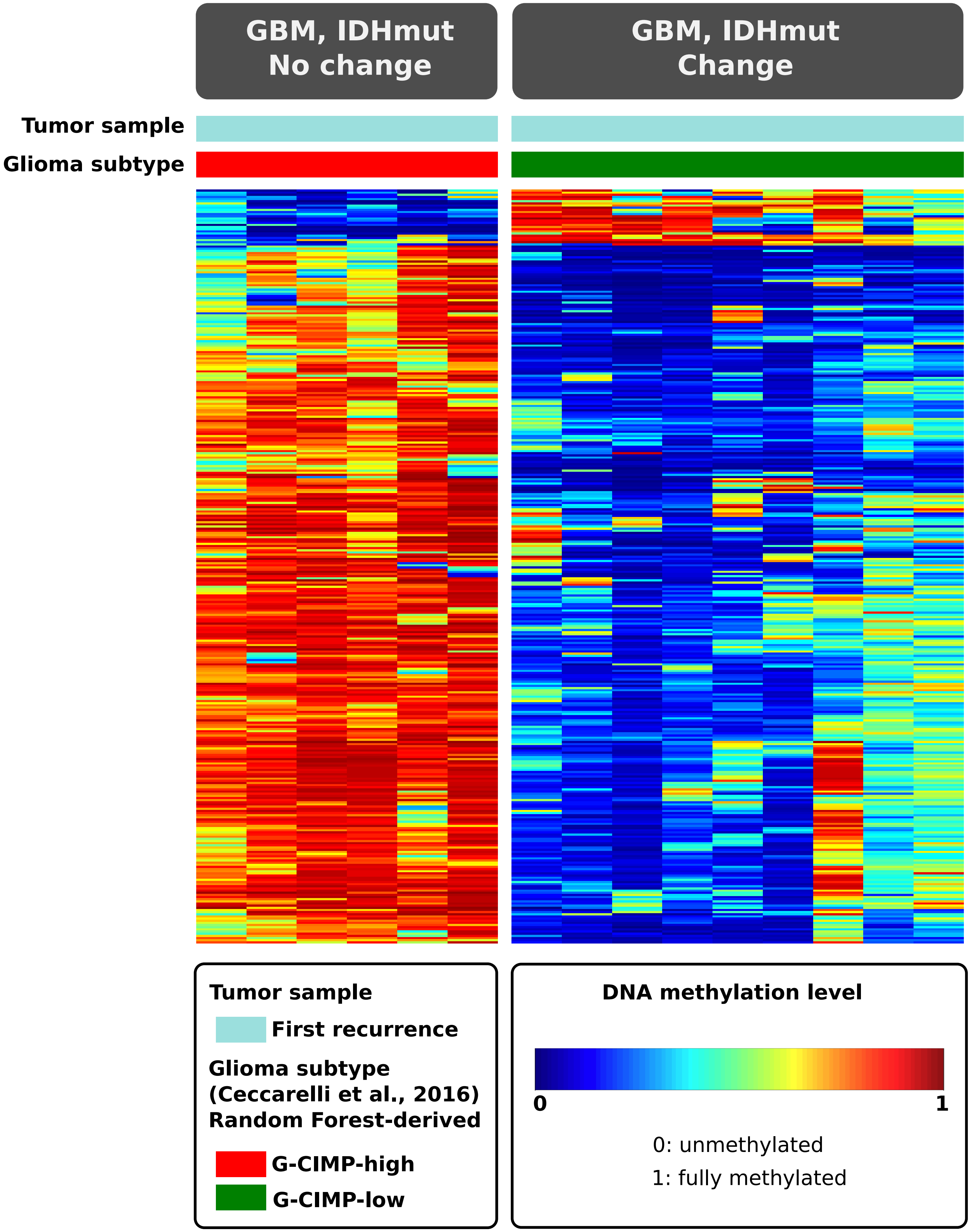

GBM, IDHmut
No change
GBM, IDHmut
Change
Tumor sample
Glioma subtype
Tumor sample
First recurrence
Glioma subtype
(Ceccarelli et al., 2016)
Random Forest-derived
G-CIMP-high
G-CIMP-low
DNA methylation level
0
1
0: unmethylated
1: fully methylated

Figure S2. Acquisition of an IDH-wildtype and stem cell-like GBM phenotype by G-CIMP-low at recurrence is independent of histopathological grade. Related to Figures 2 and 4. Heatmap of DNA methylation data. Columns represent grade IV IDH-mutant G-CIMP-high tumors at first recurrence (n=6, No change) and grade IV IDH-mutant G-CIMP-low tumors at first recurrence (n=9, Change) sorted by hierarchical clustering. For both groups (No change and Change), glioma counterparts at initial (primary) diagnosis are classified as LGG G-CIMP-high. Rows represent CpG probes, sorted by hierarchical clustering, identified as differentially methylated after supervised analysis (n=26 hypermethylated probes and n=324 hypomethylated probes in grade IV G-CIMP-low first recurrent tumors in relation to the grade IV G-CIMP-high first recurrent tumors; unadjusted $p < 0.01$, difference in mean methylation beta-value < -0.4 and > 0.5). This suggests that G-CIMP malignant transformation and progression occurs independent of grade.

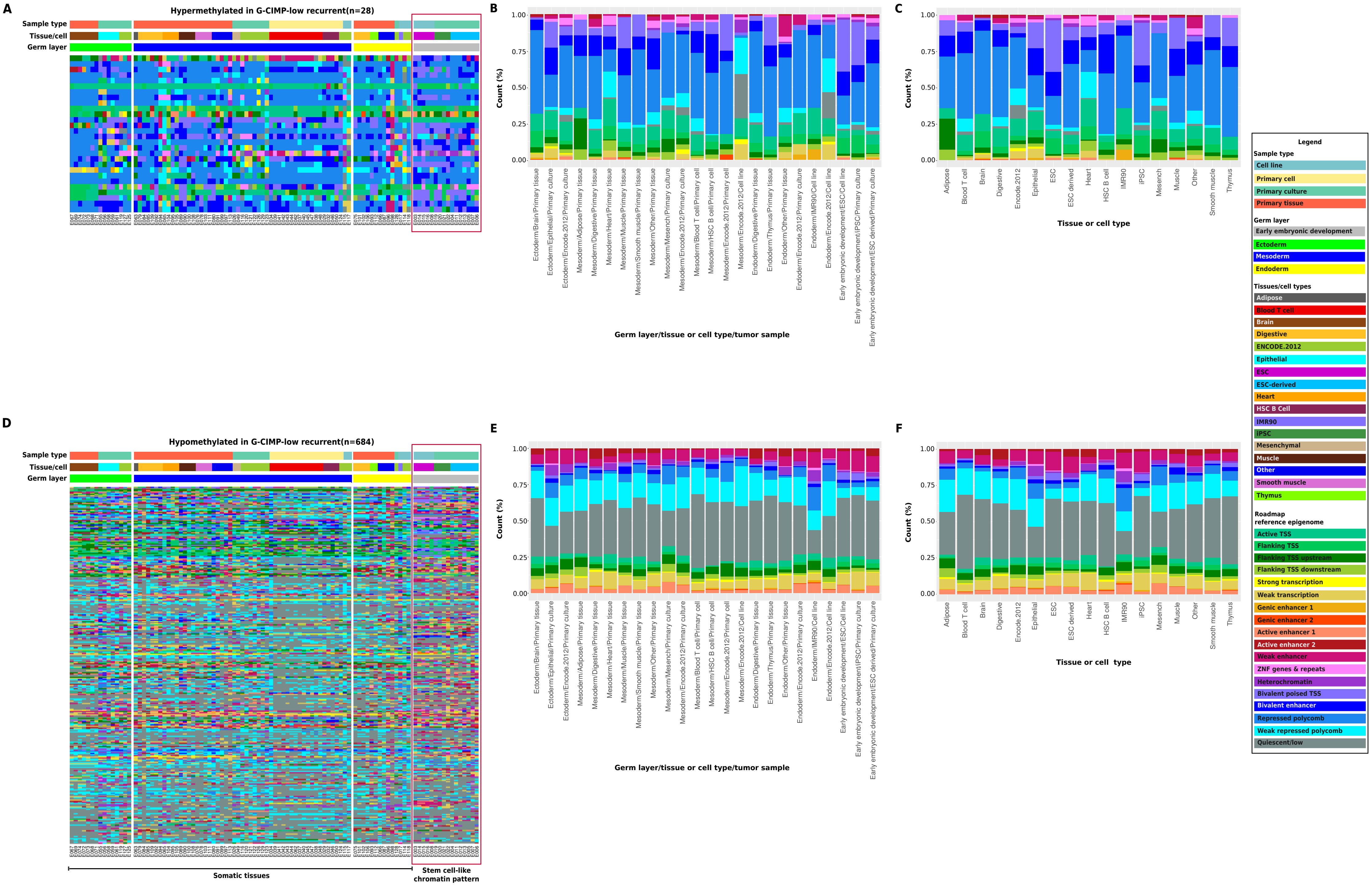
A
Hypermethylated in G-CIMP-low recurrent(n=28)
Sample type
Tissue/cell
Germ layer
B
Count (%)
1.00
0.75
0.50
0.25
0.00
Ectoderm/Brain/Primary tissue
Ectoderm/Epithelial/Primary culture
Ectoderm/Encode.2012/Primary culture
Mesoderm/Adipose/Primary tissue
Mesoderm/Digestive/Primary tissue
Mesoderm/Heart/Primary tissue
Mesoderm/Muscle/Primary tissue
Mesoderm/Smooth muscle/Primary tissue
Mesoderm/Other/Primary tissue
Mesoderm/Mesench/Primary culture
Mesoderm/Encode.2012/Primary culture
Mesoderm/Blood T cell/Primary cell
Mesoderm/HSC B cell/Primary cell
Mesoderm/Encode.2012/Primary cell
Mesoderm/Encode.2012/Cell line
Endoderm/Digestive/Primary tissue
Endoderm/Thymus/Primary tissue
Endoderm/Other/Primary tissue
Endoderm/Encode.2012/Primary culture
Endoderm/IMR90/Cell line
Endoderm/Encode.2012/Cell line
Early embryonic development/ESC/Cell line
Early embryonic development/iPSC/Primary culture
Early embryonic development/ESC derived/Primary culture
Germ layer/tissue or cell type/tumor sample
C
Count (%)
Adipose
Blood T cell
Brain
Digestive
Encode.2012
Epithelial
ESC
ESC derived
Heart
HSC B cell
IMR90
iPSC
Mesench
Muscle
Other
Smooth muscle
Thymus
Tissue or cell type
D
Hypomethylated in G-CIMP-low recurrent(n=684)
Sample type
Tissue/cell
Germ layer
Somatic tissues
Stem cell-like chromatin pattern
E
Count (%)
Germ layer/tissue or cell type/tumor sample
F
Count (%)
Tissue or cell type
Legend
Sample type
Cell line
Primary cell
Primary culture
Primary tissue
Germ layer
Early embryonic development
Ectoderm
Mesoderm
Endoderm
Tissues/cell types
Adipose
Blood T cell
Brain
Digestive
ENCODE.2012
Epithelial
ESC
ESC-derived
Heart
HSC B Cell
IMR90
IPSC
Mesenchymal
Muscle
Other
Smooth muscle
Thymus
Roadmap reference epigenome
Active TSS
Flanking TSS
Flanking TSS upstream
Flanking TSS downstream
Strong transcription
Weak transcription
Genic enhancer 1
Genic enhancer 2
Active enhancer 1
Active enhancer 2
Weak enhancer
ZNF genes & repeats
Heterochromatin
Bivalent poised TSS
Bivalent enhancer
Repressed polycomb
Weak repressed polycomb
Quiescent/low

Figure S3. G-CIMP-low CpG signatures at first recurrence discriminate between differentiated adult tissue phenotypes and pluripotent stem cells. Related to Figures 3 and 4. Integrative analysis of 98 reference human epigenomes, profiled by the NIH Roadmap Epigenomics Consortium (Roadmap Epigenomics Consortium et al., 2015), and genomic regions overlapping (A-C) hypermethylated probes (n=28) and (D-F) hypomethylated probes (n=684) that define the G-CIMP-low methylome at first recurrence. Heatmaps of chromatin states defined by dynamics of epigenomics marks not only discriminate between differentiated adult tissue phenotypes, including normal brain, and pluripotent stem cells, but also suggest a conserved mechanism between stem cell development and G-CIMP malignant transformation (epithelial to mesenchymal transition).

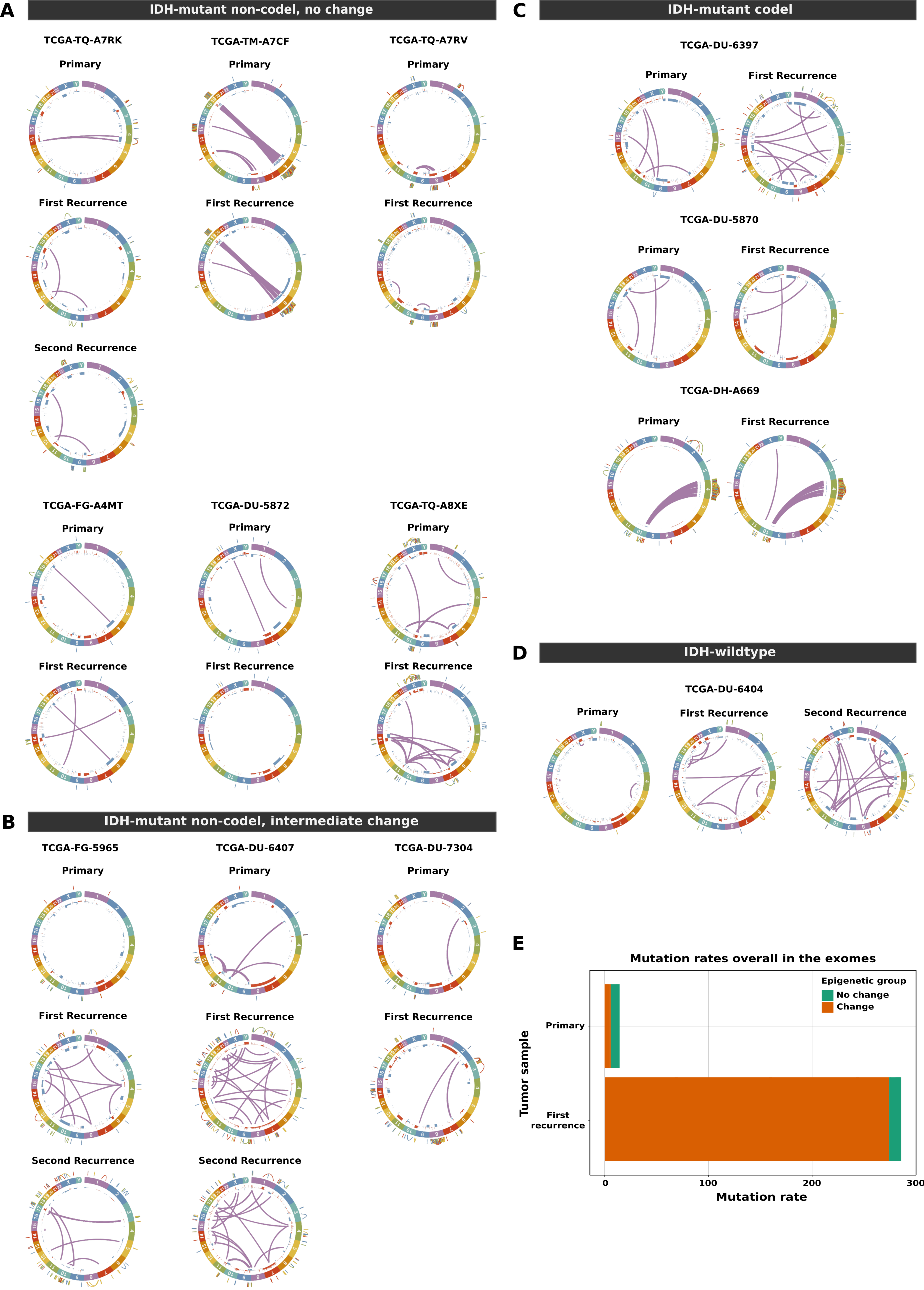
A
IDH-mutant non-codel, no change
TCGA-TQ-A7RK
TCGA-TM-A7CF
TCGA-TQ-A7RV
Primary
First Recurrence
Second Recurrence
TCGA-FG-A4MT
TCGA-DU-5872
TCGA-TQ-A8XE
B
IDH-mutant non-codel, intermediate change
TCGA-FG-5965
TCGA-DU-6407
TCGA-DU-7304
C
IDH-mutant codel
TCGA-DU-6397
TCGA-DU-5870
TCGA-DH-A669
D
IDH-wildtype
TCGA-DU-6404
E
Mutation rates overall in the exomes
Epigenetic group
No change
Change
Tumor sample
Primary
First recurrence
0
100
200
300
Mutation rate

Figure S4. Biological relationship of genomic instability and the evolution of G-CIMP methylomes. Related to Figure 2. Whole-genome rearrangement analysis across (A) initially LGG IDH-mutant non-codel G-CIMP-high tumors and their matched recurrences that retain the precursor G-CIMP-high epimethylpattern (n=6 patients and 13 tumors, No change); (B) IDH-mutant non-codel diffuse longitudinal gliomas belonging to the group that displays modest (intermediate) shift in the epigenome profiling trending towards G-CIMP-low phenotype (n=3 patients and 8 tumors, Intermediate change); (C) IDH-mutant codel (n=3 patients and 6 tumors) and (D) IDH-wildtype (n=1 patient and 3 tumors). Chromosomal rearrangements increase significantly in G-CIMP-intermediate at recurrence, resembling the genomic instability found in the IDH-wildtype glioma case at recurrence. (E) Overall mutation rate (Johnson et al., 2014) increase significantly in the exome of G-CIMP-low tumors at first recurrence in relation to their G-CIMP-high precursor counterparts (n=4 patients and 8 tumors, Change) and in relation to G-CIMP-high tumors that relapse without disease progression (n=8 patients and 16 tumors, No change), suggesting that genomic instability accumulates alongside the evolution of G-CIMP methylomes. See ‘discussion’ section.

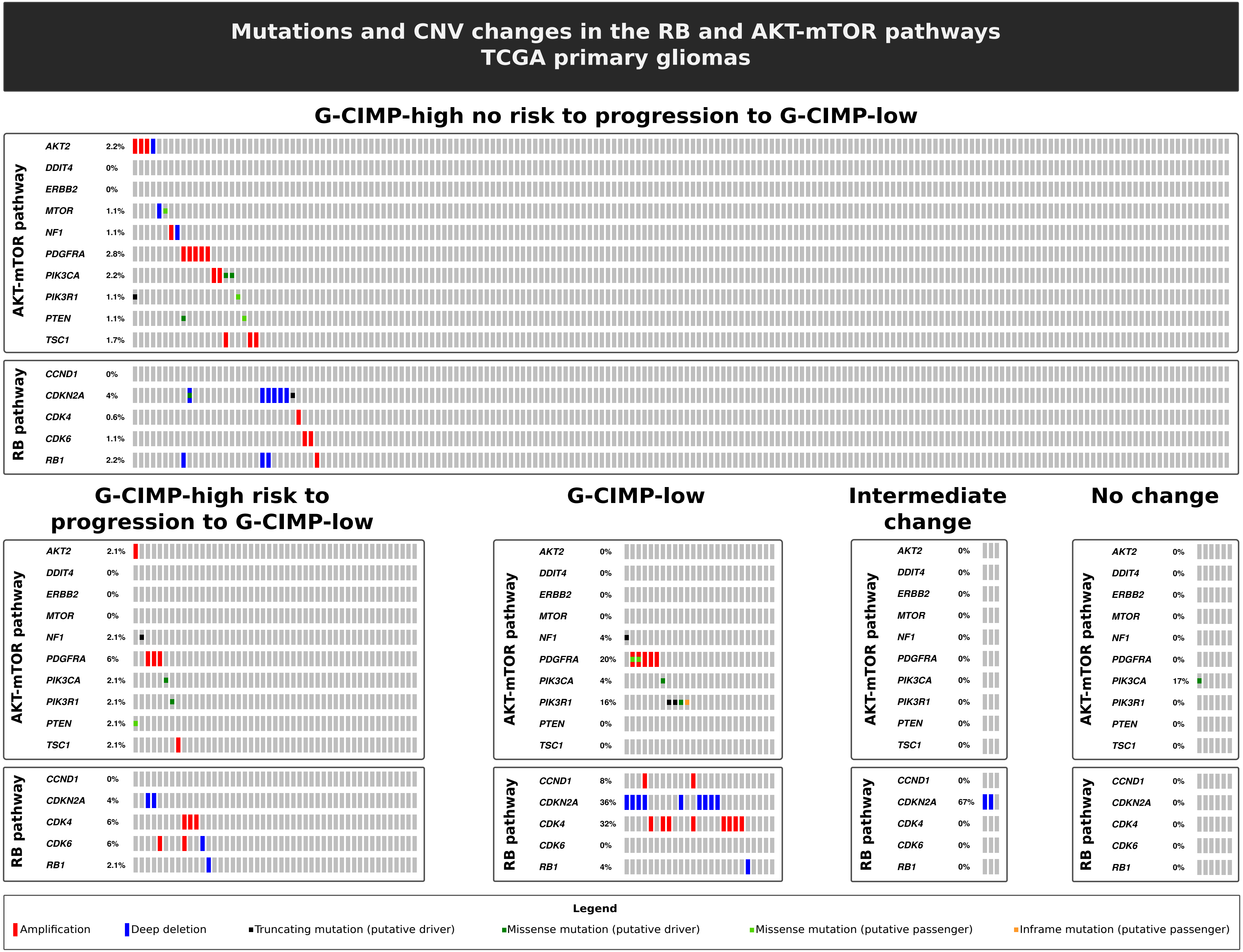
Mutations and CNV changes in the RB and AKT-mTOR pathways
TCGA primary gliomas
G-CIMP-high no risk to progression to G-CIMP-low
AKT-mTOR pathway
AKT2 2.2%
DDIT4 0%
ERBB2 0%
MTOR 1.1%
NF1 1.1%
PDGFRA 2.8%
PIK3CA 2.2%
PIK3R1 1.1%
PTEN 1.1%
TSC1 1.7%
RB pathway
CCND1 0%
CDKN2A 4%
CDK4 0.6%
CDK6 1.1%
RB1 2.2%
G-CIMP-high risk to progression to G-CIMP-low
AKT-mTOR pathway
AKT2 2.1%
DDIT4 0%
ERBB2 0%
MTOR 0%
NF1 2.1%
PDGFRA 6%
PIK3CA 2.1%
PIK3R1 2.1%
PTEN 2.1%
TSC1 2.1%
RB pathway
CCND1 0%
CDKN2A 4%
CDK4 6%
CDK6 6%
RB1 2.1%
G-CIMP-low
AKT-mTOR pathway
AKT2 0%
DDIT4 0%
ERBB2 0%
MTOR 0%
NF1 4%
PDGFRA 20%
PIK3CA 4%
PIK3R1 16%
PTEN 0%
TSC1 0%
RB pathway
CCND1 8%
CDKN2A 36%
CDK4 32%
CDK6 0%
RB1 4%
Intermediate change
AKT-mTOR pathway
AKT2 0%
DDIT4 0%
ERBB2 0%
MTOR 0%
NF1 0%
PDGFRA 0%
PIK3CA 0%
PIK3R1 0%
PTEN 0%
TSC1 0%
RB pathway
CCND1 0%
CDKN2A 67%
CDK4 0%
CDK6 0%
RB1 0%
No change
AKT-mTOR pathway
AKT2 0%
DDIT4 0%
ERBB2 0%
MTOR 0%
NF1 0%
PDGFRA 0%
PIK3CA 17%
PIK3R1 0%
PTEN 0%
TSC1 0%
RB pathway
CCND1 0%
CDKN2A 0%
CDK4 0%
CDK6 0%
RB1 0%
Legend
Amplification
Deep deletion
Truncating mutation (putative driver)
Missense mutation (putative driver)
Missense mutation (putative passenger)
Inframe mutation (putative passenger)

Figure S5. Mutational and copy-number variation (CNV) profiles of RB and AKT-mTOR pathways across a spectrum of TCGA adult diffuse primary gliomas. Related to Figures 2 and 5. G-CIMP-high and G-CIMP-low patients are stratified based on epigenomics shift profiles from primary to first recurrent diseases (when applicable) and risk to G-CIMP-low malignant transformation. Primary cases that relapse as G-CIMP-intermediate as well as primary cases classified as G-CIMP-low share genetic abnormalities (deletion) at the *CDKN2A* gene *locus* (67% [n=2 out of 3] in G-CIMP-intermediate and 36% [n=9 out of 25] in G-CIMP-low). Mutations and copy-number data were obtained from (Ceccarelli et al., 2016) and visualized using cBio portal (http://www.cbioportal.org/). See 'discussion' section.
